# Conceptualising and mapping polarisation trends in land systems

**DOI:** 10.1007/s13280-025-02344-0

**Published:** 2026-01-24

**Authors:** Christian Levers, Julian Helfenstein, Matthias Bürgi, Niels Debonne, Vasco Diogo, Rebekka Dossche, Felix Herzog, Yafei Li, Franziska Mohr, Rebecca Swart, Tim G. Williams, Peter H. Verburg

**Affiliations:** 1 Department of Environmental Geography, Institute for Environmental Studies (IVM), Vrije Universiteit Amsterdam, De Boelelaan 1111, 1081 HV Amsterdam, The Netherlands; 2 Thünen Institute of Biodiversity, Johann Heinrich von Thünen Institute - Federal Research Institute for Rural Areas, Forestry, and Fisheries, Bundesallee 65, 38116 Brunswick, Germany; 3 Geography Department, Humboldt-Universität zu Berlin, Unter den Linden 6, 10099 Berlin, Germany; 4 Agricultural Landscapes and Biodiversity, Agroscope, Reckenholzstrasse 191, 8046 Zurich, Switzerland; 5 Soil Geography and Landscape Group, Wageningen University and Research, Droevendaalsesteeg 3, 6708 PB Wageningen, The Netherlands; 6 Land Change Science Research Unit, Swiss Federal Research Institute WSL, Zürcherstrasse 111, 8903 Birmensdorf, Switzerland; 7 Institute of Geography, University of Bern, Hallerstrasse 12, 3012 Bern, Switzerland; 8 Life Cycle Assessment Research Group, Agroscope, Reckenholzstrasse 191, 8046 Zurich, Switzerland; 9 Department of Antiquity, Philosophy and History (DAFIST), University of Genova, Via Balbi 2-4-6, 16126 Genoa, Italy; 10 Soil Quality and Soil Use Research Group, Agroscope, Reckenholzstrasse 191, 8046 Zurich, Switzerland; 11 TdLab, Department of Environmental Systems Science, ETH Zurich, Universitätstrasse 16, 8092 Zurich, Switzerland; 12 Ecological-Economic Policy Modelling Research Group, University of Hohenheim, Schwerzstr. 46, 70599 Stuttgart, Germany

**Keywords:** Indicators, Landscapes, Land-use change, Multi-scale, Pathways, Social–ecological systems

## Abstract

Polarisation is a key process in land systems, describing land-change trajectories within a region that simultaneously move in opposite directions, such as co-occurring agricultural intensification and abandonment. Land-system polarisation relates strongly to sustainability goals, with potentially positive or negative consequences depending on domain and context. Yet, we lack approaches for mapping polarisation, limiting our understanding of its spatiotemporal distribution and integration into policy or impact assessments. We address this gap by exploring the concept of land-system polarisation and identifying five key dimensions for its mapping: spatial scales, temporal scales, land-use sectors, indicators, and neighbourhood relationships. We propose and test a workflow using the EU’s crop production systems, showing that ~ 87% of NUTS2 regions display polarisation. Our transferable workflow is adaptable across scales and contexts, enabling polarisation to serve as a lens for examining land-change drivers and impacts and providing spatially explicit information to guide targeted intervention and monitoring efforts.

**Supplementary Information:**

The online version contains supplementary material available at 10.1007/s13280-025-02344-0.

## Introduction

Land systems are terrestrial social–ecological systems where humans and the environment interact through land use and provide vital Nature’s Contributions to People (Ellis et al. 2019; Meyfroidt et al. 2022). Land systems are at the interface of multiple, often competing, societal demands. On the one hand, there is a global concern about how to produce sufficient food, feed, fibre, and other products for a growing and increasingly affluent world population (Erb et al. 2024). To meet this growing demand, the human use of land has increased tremendously in extent and intensity over the last decades (Winkler et al. 2021), leading to substantial changes in global land systems (van Asselen and Verburg 2013) and adverse impacts on social and ecological systems (Foley et al. 2005). At the same time, rising demand for land-based carbon sequestration, improved water regulation and purification, and biodiversity conservation are placing additional demands on already pressured landscapes (Boretti and Rosa 2019; Erb et al. 2024). Consequently, land systems are a key driver of global environmental change and themselves impacted by ongoing climate change and biodiversity crises (Verburg et al. 2015). Observing and understanding land-system dynamics is hence key to addressing current sustainability challenges (Meyfroidt et al. 2022). However, assessments of land-system dynamics can be challenging and lead to blurred or even contradictory results (Winkler et al. 2021), for example, depending on the spatial and/or temporal scale at which they play out (Gibson et al. 2000), or the selection and suitability of the indicators that are used (Rounsevell et al. 2012).

Land systems encompass interconnected biophysical, socioeconomic, and institutional components (Liu et al. 2013). Land use, which describes specific human management and modification of terrestrial surfaces, is conceptually embedded within land systems (Meyfroidt et al. 2022). Land-use change is multidimensional and context-specific, with potentially overlapping or nested processes. For example, regions might undergo changes in the *extent* of particular land uses (e.g. agriculture or forestry) and/or experience changes in management *intensity*. Examples of changing land-use extent include deforestation to facilitate agricultural expansion (Curtis et al. 2018), urban expansion over natural or agricultural areas (Liu et al. 2019), or the abandonment of agricultural areas and associated regrowth of semi-natural vegetation (Estel et al. 2015). Examples of changing land-use intensity include both intensification and de-intensification of management intensity within particular subsystems, for example, in agriculture (Levers et al. 2016). These processes can play out on different spatial and temporal scales and can potentially overlap, resulting in various changes in land-use composition and associated social-ecological impacts. Accounting for this context-specificity and multidimensionality is hence key for better understanding land-use dynamics.

Land-system polarisation exemplifies multiple, interconnected land-change processes occurring simultaneously within or between geographic regions. Generally, polarisation describes the process of division into two sharply distinct opposites (Merriam-Webster). In the scientific context, polarisation was initially used in (electro)chemistry to describe electrolysis. Nowadays, polarisation is also used to describe many other processes throughout the natural and social sciences (see Table S1 for a non-exhaustive overview of how the concept of polarisation is used in various fields). Considering the diversity of scientific fields employing the concept of polarisation, we apply their mutual and underlying prerequisite to our study: the process of division into two distinct opposites.

Land-system polarisation consequently describes land-change trajectories within or across spatial units moving in opposite directions. It is considered a key process shaping the rapid and fundamental transformations of land systems worldwide (Plieninger et al. 2014). Prominent examples of land-system polarisation dominantly address gradients of land-use extent and intensity, for instance, the regional co-occurrence of agricultural abandonment and agricultural intensification, e.g. through scale enlargement and more intensive farming on remaining agricultural lands (Plieninger et al. 2016). Although differences in biophysical conditions and social-ecological processes inherently lead to spatial variation in land systems over time, globalised markets and technological development can introduce new path dependencies that further reinforce regional specialisation and homogenisation (Jongman 2002). These dynamics have accelerated global agrifood markets and supply chains, reshaping land systems with land-change trajectories often diverging depending on to how market policies align or conflict with sustainability policies (Primdahl and Swaffield 2010).

Land-system polarisation can result from land-change processes that differ in whether polarisation itself is an intended outcome, ranging from intentional to partly intentional to unintentional. Here, intention refers specifically to whether actors driving land-change processes aim to promote or reinforce polarisation of land-use patterns. Intentional polarisation can emerge from land-sparing policies (intensification allows de-intensification elsewhere), exemplified by current Dutch policy proposals to designate an “agrarian main structure” for intensive farming and “societal agrarian areas” with reduced intensity (Bakker et al. 2022). Partly intentional polarisation can occur when shifts in intensity in a given location trigger deliberate counter-movements elsewhere, such as the rise in organic livestock farming (de-intensification) in Austria in response to dairy intensification in north-western Europe during market unification (Verburg et al. 2022). Polarisation can be an unintended outcome from market mechanisms (e.g. intensification pricing out less productive areas) or economies of scale (e.g. larger farms outcompete and absorb smaller farms), which is important to account for when developing policies to avoid undesirable consequences.

Observed polarisation trajectories can result from a single driver triggering substitution effects, or multiple drivers pushing different parts of a system in divergent directions. This poses a challenge for scientists and policy makers, as different land-change pathways can occur at different spatial scales (Meyfroidt et al. 2018), which can blur trends within target regions. For example, overall nitrogen fertiliser application may appear stable within a region, while in fact application levels are increasing in some parts (intensification) and decreasing in other parts (de-intensification). Moreover, polarisation can occur in telecoupled systems, with distant regions linked by socioeconomic and environmental interactions (Liu et al. 2013), for example, through connections between increases and decreases of feed production in disjoint regions (Silva et al. 2017). To understand the root causes and effectively address them, these differences need to be known, understood, and adequately considered.

Land-system polarisation often has substantial and diverse impacts on social-ecological systems. These impacts can be both positive and negative, depending on the target system or actor(s) affected. Moreover, depending on the time horizon of land changes, perceived impacts of land-system polarisation can differ, for example, with short-term benefits in productivity contrasting long-term decreases in Nature’s Contributions to People (Schirpke et al. 2023). Polarisation has been associated with resource use optimisation in production systems, increasing efficiency and hence lowering costs and externalities of production (Pedroli et al. 2016). However, polarisation trends have also been linked to the loss of multi-functional landscapes, mainly through increasing specialisation and concentration of land use (van der Sluis et al. 2015). Additionally, polarisation is associated with the risk of lock-ins and supply chain dependencies for land-based production systems (Conti et al. 2021), impeding sustainability transformations necessary in the light of global environmental change. It can also be linked to the decrease in rural incomes and livelihoods due to the abandonment of agricultural areas, potentially leading to the displacement of farmers (Munroe et al. 2013). Polarisation, with its opposing land-change trajectories, can potentially lead to land-use conflicts due to competition for land-based resources and trade-offs between human needs and landscape functions (García-Martín et al. 2020). Lastly, polarisation can result in land inequality, which itself can be a driver of agri-food system polarisation (Anseeuw and Baldinelli 2020).

Despite the global importance of polarisation for social-ecological systems, we lack a unifying conceptualisation of land-system polarisation (Plieninger et al. 2016). This hinders a reliable mapping of polarisation patterns, as well as the assessment of associated drivers and potential impacts on social–ecological systems. The current use of land-system polarisation in the academic literature is mostly anecdotal and descriptive and lacks conceptual depth and spatial explicitness (Antrop 2004, 2006; Primdahl et al. 2013; Plieninger et al. 2014, 2016). Existing studies that mapped polarisation trends have done so only implicitly (Levers et al. 2018) or focused on a single spatial scale and sector (Stürck et al. 2018). However, land-system polarisation is more complex as it can occur on different spatial (e.g. farm, landscape, regional, global) and temporal (short term to long term) scales (Plieninger et al. 2016), both within (e.g. cropland abandonment and cropland intensification) and between (e.g. cropland abandonment and livestock intensification) land-use sectors (Plieninger et al. 2016), and pertain to a variety of indicators (e.g. extent, intensity, or spatial patterns). So far, no study has consistently and comprehensively incorporated these features to conceptualise and map land-system polarisation. This knowledge gap requires attention because better understanding polarisation patterns can help to assess its drivers and outcomes, as well as to avoid misinterpretations of land-system dynamics. Further, identifying regions undergoing land-system polarisation would allow spatially explicit assessments of its potential trade-offs and the option space to navigate them. Knowledge on land-system polarisation thus helps widen our understanding of land-system dynamics and could support decision-makers in designing targeted policies and measures.

Here, we present and apply an analytical framework that provides a unified and structured workflow for characterising and mapping land-systems polarisation. Specifically, we (1) define system boundaries of polarisation, as well as key dimensions and indicators that can be used to map land-system polarisation, (2) develop a workflow to map land-system polarisation and apply it to a real-world example (EU’s crop production systems), and (3) discuss how maps of land-system polarisation can help to better understand land-system dynamics and potentially support policy making. We demonstrate that mapping land-system polarisation provides a powerful lens to analyse complex land systems. It allows a more nuanced understanding of land dynamics compared to traditional land-use change mapping by combining different land-change types and trajectories (e.g. changes in intensity or extent), which are commonly addressed individually (e.g. intensification or abandonment), under a common analytical umbrella. Moreover, it allows identifying regions prone to potential (sectorial) conflicts (e.g. regions with increasing nature-conservation efforts coinciding with intensifying and expanding agriculture). Mapping polarisation can hence initiate broader discussions about their consequences and provide important insights for decision-making in contexts where complex land-system dynamics occur. While our practical example focuses on land-use changes, we use the term ‘land-system polarisation’ throughout the manuscript as land-use change is a central component of land-system change (Turner et al. 2021). Moreover, our adaptable, scalable, and transferable conceptualisation and analytical framework are not limited to land-use change; they can be extended to encompass additional dimensions of land systems if adequate spatial data are available.

## An analytical framework for mapping land-system polarisation

**Fig. 1:**
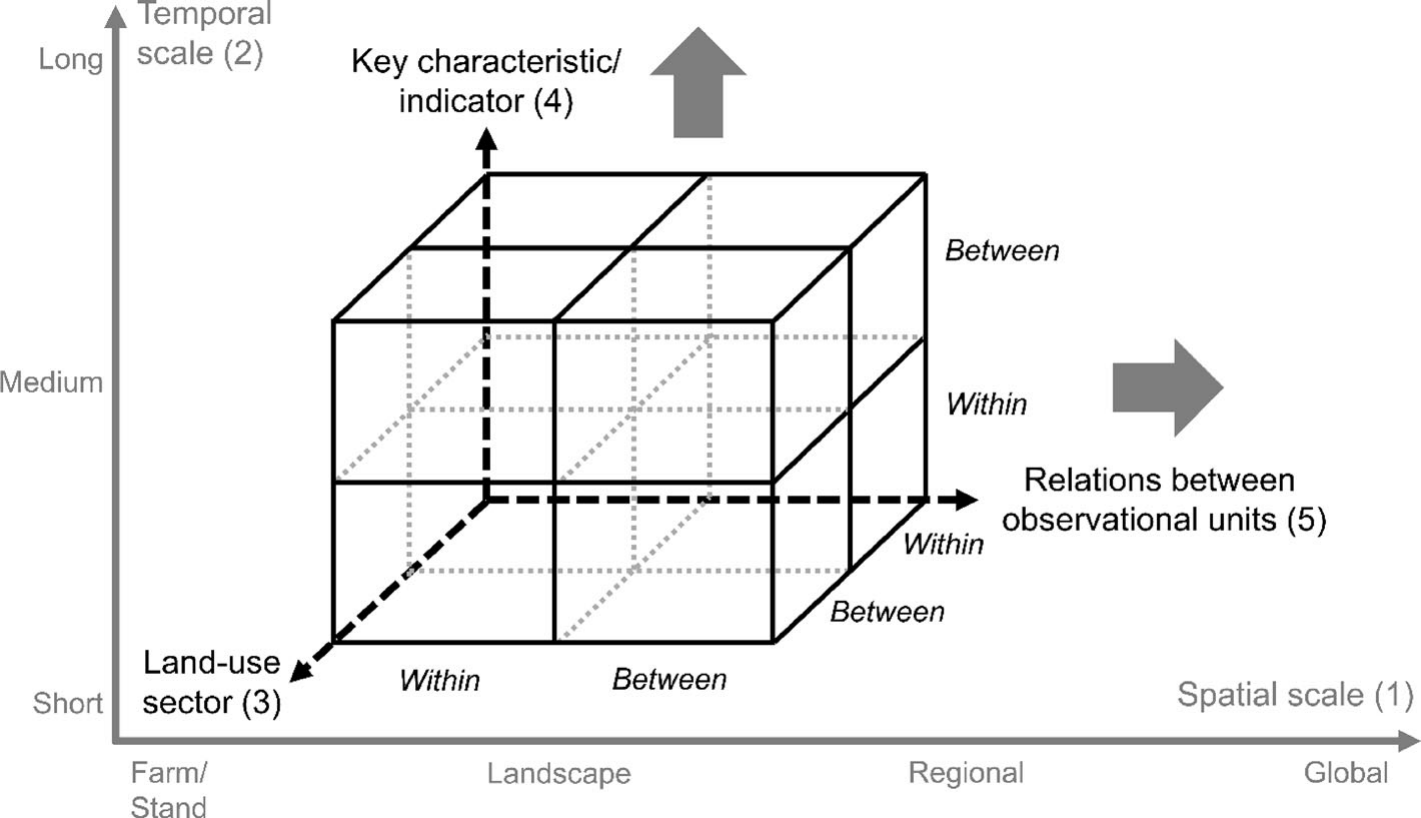
Schematic overview of polarisation dimensions. Relationships between observational units are displayed on the x-axis of the cube (5), key characteristics and related indicators on the y-axis of the cube (4), and land-use sectors on the z-axis of the cube (3), positioned along spatial (1) and temporal (2) gradients of the Cartesian coordinate system (x-axis and y-axis, respectively). Analyses of polarisation take a fixed spatiotemporal scale (i.e. point in the Cartesian coordinate system) and assess diverging trends within or between the three other dimensions (i.e. quadrants in the cube)

We conceptualise land-system polarisation as a scale- and unit-dependent, spatially explicit, and quantifiable/observable process that involves diverging trajectories between or within interconnected land-system dimensions. Hence, data used for mapping polarisation need to be available in a spatially explicit form, i.e. either as raster or vector data, and at different points in time (e.g. two target years or a time series). Importantly, our concept captures the outcomes of decision-making on observable land-system properties, which can be affected by potential polarisation of intangible dimensions such as values or beliefs (Fig. S1), and mediated by time lags between decisions and outcomes (Brown et al. 2019). Expanding on Plieninger et al. (2016), we propose five dimensions relevant for mapping land-system polarisation (Fig. 1): (1) spatial scales, (2) temporal scales, (3) land-use sectors, (4) key characteristics/indicators, and (5) relationships/links between observational units.

Land-system polarisation can hence be understood as a complex, multi-dimensional phenomenon, which can occur from local to global scales and can extend over short to long time periods (Fig. 1). Although polarisation could potentially occur between spatial scales (e.g. national-scale agricultural intensification co-occurring with landscape-scale agricultural abandonment) or temporal scales (e.g. long-term agricultural abandonment co-occurring with short-term agricultural intensification), it is difficult to establish a relationship between such polarisation processes given differences in observational units, their potential drivers, and different temporal baselines and time horizons. Mapping land-system polarisation at fixed spatiotemporal scales and combining the resulting assessments at different spatial and/or temporal scales allows identifying nested polarisation trends. For example, a region might undergo polarisation characterised by agricultural abandonment and intensification occurring simultaneously in different sub-regions, while it is embedded in a country experiencing the same or different polarisation trends.

At a given spatiotemporal scale, polarisation can occur within or between the remaining dimensions. Regarding land-use sectors (“Land-use sectors” section), polarisation can describe simultaneous cropping intensity increase and decrease (within-sector), or cropping intensity increase and grazing intensity decrease (between-sector). Regarding key characteristics and indicators (“Key characteristics and indicators” section), polarisation can describe simultaneous cropland area increase and decrease (within-indicator) as well as cropping intensity increase and cropland area decrease (between-indicator). Regarding observational units (“Relations between observational units” section), polarisation can describe simultaneous area increase and area decrease within the same observational unit (within-unit) as well as area increase in one and area decrease in another observational unit (between-unit), with regions linked through trade, for example. Importantly, to avoid potential dependencies of land-change processes (e.g. decreased crop extent could lead to increased crop yields on a farm, if the area out of production has low agronomic potential), we conceptualise land-system polarisation as a temporally parallel, but spatially disjoint process. Hence, data aggregation (e.g. farms/stands to landscapes) might be necessary if it cannot be ruled out that increasing and decreasing trends (here: intensity and area changes) spatially co-occur.

By focussing on quantifiable and mappable features of land systems and their management, we thereby leave out intangible features of land-system polarisation, which can be important elements for a comprehensive assessment of polarisation, such as polarisation in belief and value systems of land managers (Klebl et al. 2024). This is partly due to the fact that quantitative data representing such intangible features are generally scarce and difficult to map (Otto et al. 2015). However, our analytical framework can be adapted to include additional key characteristics and indicators of land-system polarisation if they are quantifiable and mappable. Our analytical framework further leaves aside any normative evaluation of polarisation due to the strong dependence on positioning, belief system, and context-dependency of outcomes (Meyfroidt et al. 2022). For example, polarisation can have negative connotations from a conservation perspective (e.g. loss of agro-biodiversity), but positive connotations from a value chain perspective (e.g. higher efficiency and revenues) for co-occurring agricultural abandonment and intensification in a given region. However, narratives of polarisation itself can play a strong role in land-system governance, as opposing land-system visions and positions, for example, by different stakeholders in decision processes over land, could result in land-system polarisation. Lastly, we highlight that not all opposing land-change trajectories represent polarisation, only those which are interconnected or interdependent. Clearly defining the target system, system boundaries, observational units, and indicators of interest is hence key for mapping polarisation, as outcomes are only interpretable within such contexts and can mislead otherwise.

### Spatial and temporal scales

We propose four spatial scales as observational units relevant to land-system polarisation, each relating to key organisational scales for policy- and decision-making (Diogo et al. 2022): (1) farm/forest management unit scale (i.e. management unit in agriculture/forestry), (2) landscape scale (i.e. biophysical unit characterised by social–ecological features), (3) regional scale (i.e. administrative unit, including sub-national regions, countries, or supra-national regions), and (4) global scale (i.e. bounded by the Earth system). The farm/forest management unit constitutes the smallest observational scale to map land-system polarisation. Land management decisions and activities at this scale (usually at the field/stand level) can be split between land users (day-to-day management) and land owners (longer-term decision-making), as these are often different persons or entities (e.g. the EU had nearly as much rented farmland as owned farmland in 2020; (Eurostat 2024)). Landscape and regional scales are key target scales for policy making to address sustainability challenges (Wu 2013), with landscapes representing spatially distinct units with characteristic patterns, functions, and dynamics resulting from the interplay of ecological processes and human use of land. Polarisation on global scales can indicate potential imbalances and trade-offs of land-system dynamics.

Land-change processes occur over varying time scales, ranging from short-term changes that cover time frames of a few years (or in specific cases even less than a year) to long-term changes that cover time frames of decades to centuries (Watson et al. 2014). Contrary to spatial scales, it is difficult to define distinct time intervals for land-system polarisation, as processes can have varying speeds due to different land-change types, site characteristics, and actors involved. Annual processes occur within a single calendar or growing year (e.g. seasonal crop shifts), short-term processes span few years (~ 2–5) as immediate responses to triggers (e.g. policy changes, price spikes, early technology diffusion), medium-term processes span multiple years (~ 5–20) but within human decision-making horizons (e.g. forest rotations, farm restructuring), long-term processes span multiple decades (~ 20–50 years, e.g. forest succession, desertification, rural depopulation), while centennial-scale processes encompass intergenerational or historical transitions (~ 50–100 + years, e.g. land changes resulting from industrial revolution and colonialism). Furthermore, path dependency and legacy effects of historic land use (Meyfroidt et al. 2022) complicate the definition of clear temporal scales for polarisation.

### Land-use sectors

Land systems comprise several land-use sectors, resulting in specific social–ecological system configurations. These are broadly summarised under the term AFOLU: agriculture, forestry, and other land use such as natural vegetation or built-up land (IPCC 2014). These broad classes can be further refined, e.g. agriculture into cropland and pastures, croplands into arable crops and horticulture, and arable crops into individual crop types. Polarisation often leads to spatial concentration or segregation of sectoral activities, e.g. concentration of one sector in a particular region, or one sector outcompeting others within a region, leading to their displacement to other regions (Meyfroidt et al. 2020).

### Key characteristics and indicators

**Fig. 2:**
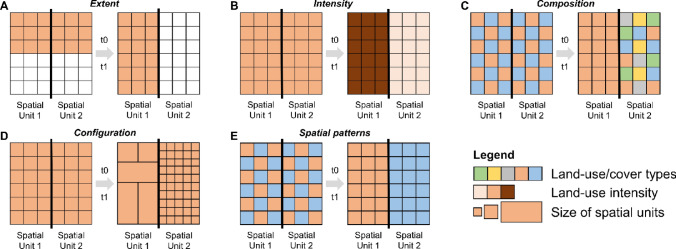
Schematic illustration of the suggested five key characteristics of polarisation: extent (**A**), intensity (**B**), composition (**C**), configuration (**D**), and spatial patterns (**E**). Each large square represents an observational unit with a thick vertical line indicating spatially disjoint sub-regions (e.g. municipalities within a province or farms/forest management units). Small squares represent operational units, e.g. fields/stands. Each panel displays the situation for two time steps: t0 (left) and t1 (right). The change from t0 to t1 (indicated by the grey arrow) represents polarisation for the respective key characteristic. Importantly, we here show key characteristics of polarisation within observational units. Location is important for polarisation processes, and hence, polarisation for each proposed key characteristic inherently includes location changes

Expanding the common approach for mapping polarisation (changes in extent and intensity), we propose five key characteristics of land systems that can indicate polarisation trends in a spatially explicit way: (1) extent, (2) intensity, (3) composition, (4) configuration, and (5) spatial patterns of the target system. Each key characteristic can be represented by various indicators (Table 1, Fig. 2). As key characteristics and indicators strongly depend on study area, scale of analysis, and data availability, we highlight that our proposition provides a starting point that can be adapted and tailored to case study specific contexts. This could include adding indicators of the social domain of polarisation, if spatially explicit time series data on variables such as age, income, or behaviour is available (see Fig. S1).

**Table 1:** Key characteristics and candidate indicators to map land-system polarisation, using crop production systems as an illustrative example. The list of indicators is exemplary and not exhaustive. Mapping indicators at one selected spatial scale, e.g. farm or landscape scale, ensures consistency and comparability

Key characteristic	Indicator	Unit	Description
Extent	Area	ha	Physical area of land-use type
Intensity	Consumable inputs	kg ha^−1^, € ha^−1^, J ha^−1^	Agricultural inputs (e.g. nitrogen and pesticides) to support crop production; intermediary inputs (e.g. fuel, energy) into production systems
	Fixed capital assets	€ ha^−1^, #tractors ha^−1^, mechanisation index	Use of machinery and equipment for crop production (mechanisation)
	Irrigation and drainage	% area irrigated, m^3^ ha^−1^, km channels km^−2^	Water regulation through irrigation or drainage systems to support crop growth and manage water conditions
	Output	kg ha^−1^, calories ha^−1^, € ha^−1^	Net output and monetary value of agricultural production per area
	Labour	annual working units ha^−1^	Amount of labour input into production systems
Composition	Diversity/specialisation	Shannon div. [0,∞], Simpson div. [0,1], Dissimilarity index [0,1]	Indicator of dominance or evenness of land-use types (e.g. cropland or grassland) or within land-use types (e.g. crop types or tree species)
	Production system	#	Number of different land-use, crop, or livestock types
Configuration	Farm size	ha	Physical area of farm or holding
		ESU (European size unit)	Economic size of farm or holding as standard gross margin of € 1200
	Field size	ha	Physical area of field plot
	Contagion	%	Aggregation or clumping of management units
	Edge density	m ha^−1^	Heterogeneity of management units
Spatial patterns	Spatial autocorrelation	Moran’s I [− 1,1], Geary’s C [0,1]	Degree of spatial clustering (from dispersed to clustered) of key characteristics/indicators

*Land-use extent* represents the spatial footprint of each land-based production system. Each land-use type has specific impacts on social–ecological systems, and changes in the extent of a certain type (i.e. expansion or contraction) usually result in an increase or decrease of such impacts, depending on the direction of change (Kuemmerle et al. 2016). We define polarisation in *extent* as a simultaneous area increase and decrease of a certain land use in spatially disjoint locations (Fig. 2A), e.g. cropland expansion due to increased production goals and cropland loss due to abandonment.

*Land-use intensity* represents the level of inputs, outputs, and system-level outcomes of land use (Erb et al. 2013). It is a complex and multidimensional phenomenon (Diogo et al. 2022), and social–ecological system impacts are usually characterised along gradients of individual intensity indicators (or combinations thereof). For example, higher land-use intensity (or intensification) is often related to higher social–ecological impacts and rarely to co-benefits with ecosystem service outcomes (Rasmussen et al. 2018). We define polarisation in *intensity* as a simultaneous increase and decrease of one or more land-use intensity indicators in spatially disjoint locations (Fig. 2B), e.g. an increase in nitrogen inputs to boost crop yields and a decrease in pesticide inputs due to management change (e.g. agri-environmental schemes, integrated pest management).

*Composition* represents the (non-spatial) diversity and frequency of land-use types within an observational unit (e.g. farm or landscape), but can also represent the diversity and frequency within a given land-use type, for example, the diversity of crop types, livestock species, or tree types. A higher diversity, for example, in mosaic landscapes, is often related to lower environmental pressures (Abson et al. 2013) and higher resilience of production systems (Helfenstein et al. 2022). Diversification strategies can simultaneously benefit social and environmental outcomes of land use (Rasmussen et al. 2024). We define polarisation in *composition* as a simultaneous increase and decrease in composition elements in spatially disjoint locations (Fig. 2C), e.g. an increase and decrease in the number of crop types, or a change of mixed arable land into arable monoculture and agroforestry.

*Configuration* in land-use types represents the size and spatial heterogeneity of management units on which land-use decisions play out (e.g. farms or forest management units). The size of management units can be linked to environmental, economic, and human wellbeing (Altieri et al. 2017), and knowledge about size distributions can foster the development of actions to address land-system sustainability (Herrero et al. 2017). We define polarisation in *configuration* as a simultaneous size increase and decrease in management units in spatially disjoint locations (Fig. 2D), e.g. increasing and decreasing farm size (Debonne et al. 2021b). Globally, polarisation in *configuration* is evident in the scale transition of agriculture: large farms tend to expand in high-income countries, while small farms tend to shrink in middle- and low-income countries (Lowder et al. 2016).

*Spatial patterns* represent the spatial arrangement of key characteristics/indicators themselves. For each key characteristic, polarisation trends entail changing locations and hence changing spatial patterns. Within a given observational unit, this can lead to completely uniform/dispersed, randomly distributed, or a completely clustered spatial arrangement of indicators. Clustered patterns indicate a spatial concentration of the respective indicator (e.g. intensifying crop production systems), while dispersed patterns indicate a more even distribution of the respective indicator across the area. Such clustering and dispersion can be measured using indicators like Moran’s I, which describes the degree of spatial autocorrelation of a variable (e.g. fertiliser use intensity) within a region of interest. We define polarisation in *spatial patterns* as a simultaneous clustering and dispersion of a key characteristics within a region (Fig. 2E). A prominent example for this is the land sparing concept, which aims to reconcile food production and biodiversity conservation by intensifying and concentrating production in highly productive areas, thereby freeing up space for unfarmed habitats through decreases in agricultural area (Phalan et al. 2011).

### Relations between observational units

While examples of polarisation for land-use sectors (“Land-use sectors” section) as well as key characteristics and indicators (“Key characteristics and indicators” section) address the situation for polarisation trends *within* the same observational unit, polarisation trends can also occur *between* observational units. For example, cropland extent can increase in one region, while at least one ‘related’ region can be characterised by a decrease in cropland extent. Relationships between observational units can be established either by adjacency (i.e. by sharing a physical border), or through trade relationships or other forms of (non)material exchange between observational units (referring to the telecoupling concept; Liu et al. 2013). For example, units with strong trade links can be considered related despite lacking a shared border and thus may undergo polarisation. Quantifying polarisation between observational units can be difficult as it requires assumptions about the degree of ‘relatedness’ (e.g. adjacency or exchange), the importance of the ‘neighbourhood’ effect relative to other factors influencing land use (e.g. population growth), and the strength of the connection between the processes occurring in the different regions.

## Applying the framework: mapping polarisation in EU’s croplands

**Fig. 3:**
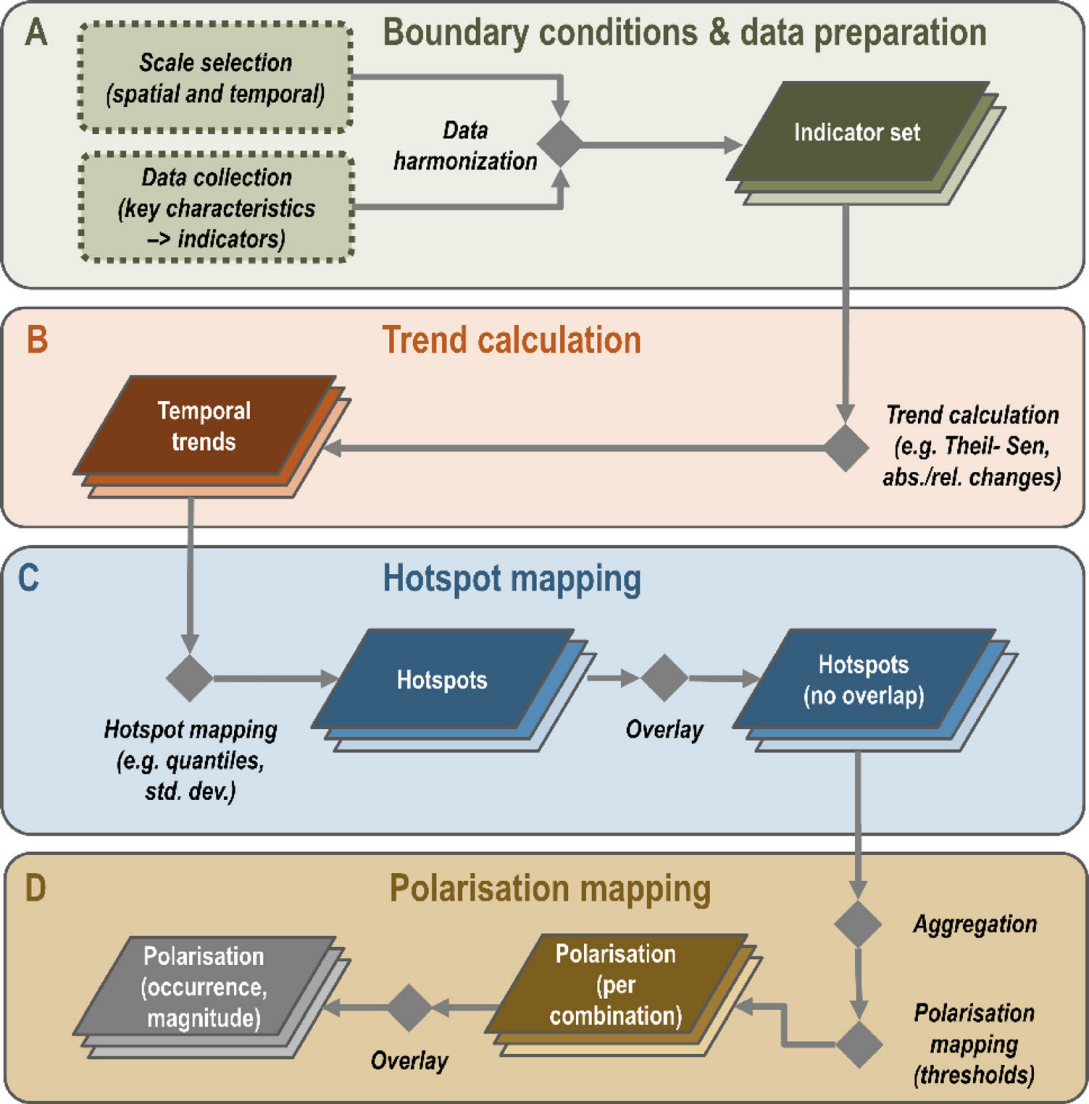
Flow diagram of key analysis steps for mapping polarisation in land systems: Defining boundary conditions and data preparation (**A**), trend calculation (**B**), hotspot mapping (**C**), and polarisation mapping (**D**)

We present a workflow for mapping land-system polarisation (Fig. 3, see Fig. S2 and Text S1 for details). The workflow consists of four key building blocks facilitating the mapping process, which can be adapted and expanded if necessary: define boundary conditions, and collect and harmonise input data (A), calculate indicator trends (B), map hotspots of change, i.e. increase and decrease (C), and map polarisation trends based on the spatial extent of change hotspots (D). We illustrate our workflow by mapping polarisation in the EU’s crop production systems using 2000–2012 data from the CAPRI model, an economic model developed through European Commission funding to support decision-making related to the Common Agricultural Policy (Britz and Witzke 2014).

We implement our workflow for the following system boundaries, largely guided by data availability and favouring simplicity over completeness. First, we select a medium temporal scale (12-year period) and a regional spatial scale (NUTS-2 units; Nomenclature des Unités territoriales statistiques) due to its relevance for decision-making and land-use planning. Second, we select crop production systems as our target land-use sector. Third, we use CAPRI-based indicators representing four key characteristics: cropland area, nitrogen input, inverse Shannon crop diversity, and spatial autocorrelation for each indicator (Table 2).

**Table 2:** Overview of indicators used for mapping polarisation in crop production systems in the EU between 2000 and 2012. We provide study-area wide mean and standard deviation for each indicator for the first (2000) and last (2012) year of our study period. Note that polarisation in spatial patterns of key characteristics can only be calculated at the next highest aggregation level (here: NUTS-2 instead of pixel-level)

Key characteristic		Indicator	Unit	Spatial resolution	Temporal resolution	Mean [S.D.] (2000)	Mean [S.D.] (2012)	Source
Extent		Cropland area	1000 ha	10 × 10 km2	2000–2012 (2-year intervals)	4.57 [3.48]	4.42 [3.48]	CAPRI
Intensity		Nitrogen input	kg/ha	10 × 10 km2	2000–2012 (2-year intervals)	83.41 [58.17]	84.99 [66.79]	CAPRI
Composition		Shannon crop diversity [0,∞]	–	10 × 10 km2	2000–2012 (2-year intervals)	1.35 [0.59]	1.38 [0.62]	CAPRI
Spatial pattern	Extent	Moran’s I [− 1,1]	–	NUTS-2	2000–2012 (2-year intervals)	0.26 [0.17]	0.27 [0.17]	Own calculation
	Intensity					0.41 [0.18]	0.42 [0.19]	
	Composition					0.29 [0.17]	0.31 [0.19]	

We use the inverse Shannon crop diversity to ensure a thematically consistent interpretation of indicator trajectories, with increasing values for extent (i.e. enlargement), intensity (i.e. intensification), and composition (i.e. specialisation or simplification) indicating more industrialised systems, and decreasing values for extent (i.e. contraction), intensity (i.e. de-intensification), and composition (i.e. diversification) indicating more extensive systems. CAPRI data are provided in raster format at a spatial resolution of 10 × 10 km^2^, (downscaled to Farm Structure Units (FSU) at 1 × 1 km^2^, which offers information about the spatial patterns within each observational unit (NUTS-2 region). Unfortunately, the CAPRI data do not provide information regarding farm size or other configurational factors for our study period.

We map polarisation *within* observational units (i.e. NUTS-2 regions), *within* and *between* indicators, and of their *spatial patterns* (see Table S3 for a summary of polarisation trajectories using examples and hypothesised mechanisms for EU cropping systems). We omit potential polarisation arising from telecoupled crop production systems. We define polarisation for a given NUTS-2 region if opposing processes (i.e. hotspots of increase and decrease of a given indicator combination, based on top and bottom quintiles of the respective data distributions) each cover an area share of > 5%. We test the sensitivity of results using a more liberal (2.5%) and conservative (10%) threshold. We assess polarisation using all grid cells of the input indicators that indicated the presence of cropland.

**Fig. 4:**
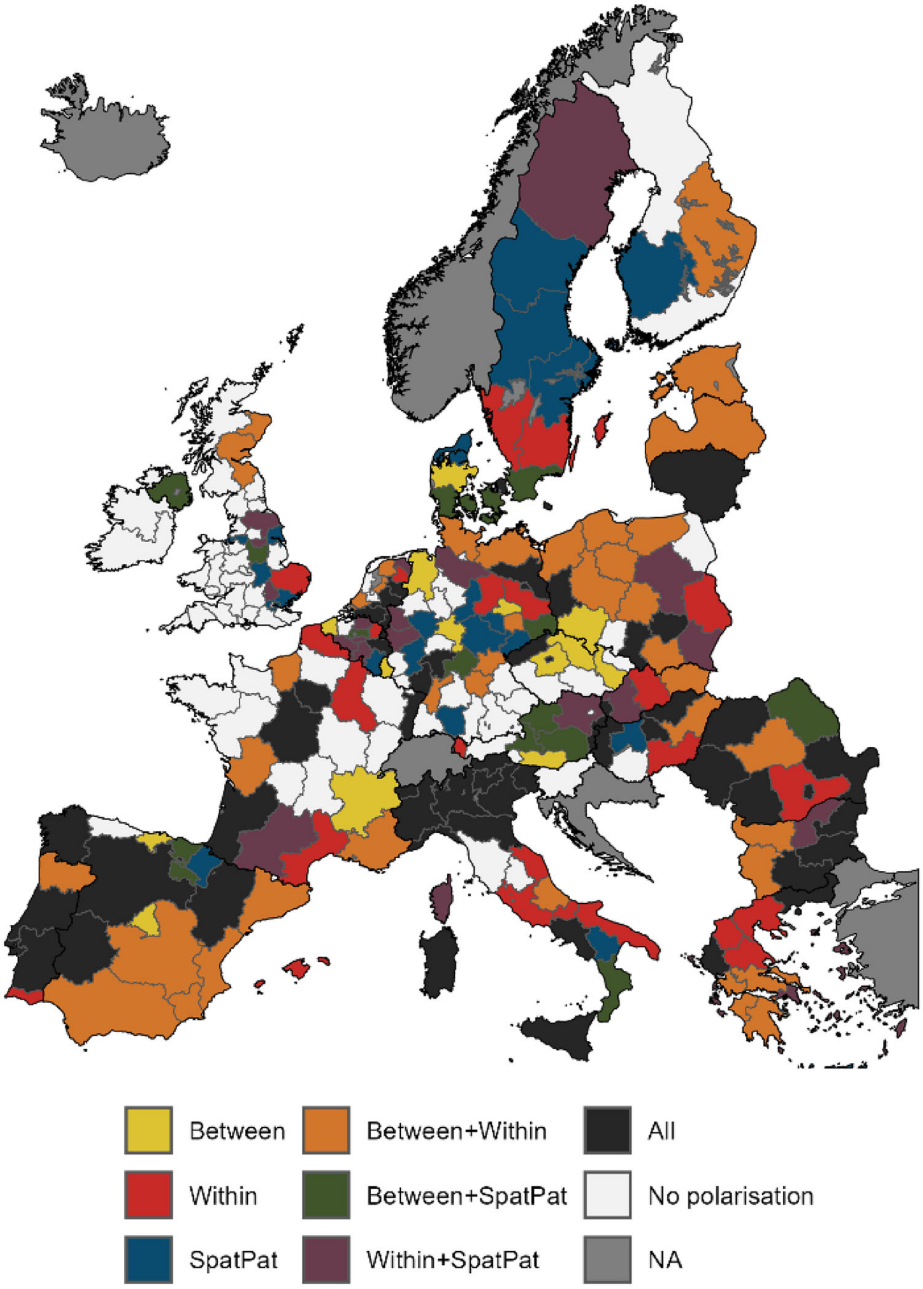
Spatial patterns of land-system polarisation at NUTS-2 level for EU’s crop production systems between 2000 and 2012. Polarisation patterns are based on a 5% threshold regarding hotspots of change, i.e. hotspots of increase and decrease each have to cover at least 5% of the area of a NUTS-2 region to indicate polarisation

Polarisation in crop production systems is a widespread process in the EU (Fig. 4, Table S2). We identify polarisation in 228 out of 261 NUTS-2 regions (~ 87%). The most widespread polarisation profile is “All”, which indicates co-occurring polarisation trends between indicator pairs, within indicators, and in its spatial pattern. This profile occurs in 90 NUTS-2 regions, mostly located in the Mediterranean and Eastern Europe, as well as Central Europe. Co-occurring polarisation between indicator pairs and within indicators is the second most widespread polarisation profile (87 NUTS-2 regions), which occurs throughout the EU, mostly in Southern and Eastern Europe. We do not find polarisation trends for 33 NUTS-2 regions (~ 13%) based on our indicators. The regions without polarisation trends are mostly located on the British Isles as well as in France and Germany.

The choice of the area threshold to map polarisation for NUTS-2 regions consequently influences the spatial patterns of polarisation in crop production systems (Figs. S3–S6). More conservative thresholds (i.e. a larger area share of a given NUTS-2 region required to be classified as a polarisation hotspot) generally lead to less widespread polarisation with fewer NUTS-2 regions showing polarisation trends. Moreover, polarisation profiles can change as a result. For example, several NUTS-2 regions on the Iberian Peninsula are characterised by polarisation profile “All” for the liberal hotspot threshold (Fig. S3A). Increasing the required area share of polarisation hotspots changes the profile for some regions into “Within + SpatPat” and “SpatPat” for the intermediate threshold (Fig. S3B), and further to “Within + SpatPat”, “SpatPat”, and “Between + SpatPat” (Fig. S3C).

Breaking down general polarisation profiles into individual polarisation types reveals specific polarisation geographies (Figs. S4–S6). For example, polarisation between cropland area and crop diversity (Fig. S5D) occurs in three forms on the Iberian Peninsula: with area increase and diversity decrease mainly in the southern part, with area decrease and diversity increase in the north-western and -eastern part, and in both directions in the south-western part. Moreover, area decrease and diversity increase are the most widespread type of this polarisation between indicators, mainly located in Eastern and Southern Europe. For the majority of polarisation types, we observe spatial clustering as neighbouring regions often exhibit the same polarisation type.

## Discussion

Land-system polarisation is an important process in social–ecological systems. Unfortunately, knowledge on where land-system polarisation occurs is scarce because we lack approaches for mapping polarisation processes. Here, we propose and document an analytical framework and a workflow for mapping land-system polarisation trends across spatiotemporal scales and apply it to crop production systems in the EU.

### Implications for science and policy

Several theoretical and practical implications arise from our work. Mapping polarisation can start a discussion on the consequences of these processes and assist the design of targeted policies to regions where these processes are having negative implications. Different polarisation trends (see Table S3 for examples) can have specific social–ecological impacts with uneven outcomes affecting land productivity, ecosystem health, and rural livelihoods. For example, agricultural intensification or specialisation may increase vulnerability to social–ecological shocks. Similarly, polarisation within crop fertilisation could indicate regions experiencing over- and under-fertilisation, potentially leading to environmental pollution alongside diminished productivity in nutrient-deficient areas (Vitousek et al. 2009). By mapping and characterising polarisation profiles, i.e. characteristic combinations of polarisation trends, entry points for context-specific decision-making can be identified that account for the interlinked dimensions of opposing trends in land systems in an integral manner.

Polarisation is a useful lens for studying complex land systems. Land-change pathways often overlap within regions (Helfenstein et al. 2024). By explicitly considering relationships among, and the co-evolution of, different land-change processes, our analytical framework is particularly well-suited to characterise compensatory effects and assess trade-offs and spillover effects of land change, both across space (e.g. between different regions) and across factors of production (e.g. between input intensity and crop diversity). Comparable to similarity or transferability analyses (Diogo et al. 2023), our structured approach to conceptualise and identify land-system polarisation allows cross-comparisons between polarisation studies, which provides a stepping stone for identifying common trends and patterns across different contexts, and synthesising knowledge towards theories of land system polarisation and their outcomes (Diogo et al. 2023). Notably, our mapped polarisation patterns coincide well with hotspots of agricultural change in Europe under alternative value perspectives (Diogo et al. 2025), with scenarios indicating divergent trajectories of land change. Combining analyses on past and future polarisation trends allows a detailed look at potential path dependencies, legacy effects, and lock-ins of land-system dynamics (Meyfroidt et al. 2022).

Land-system polarisation provides a novel lens to capture and characterise the divergence and clustering of land-use patterns. It thereby offers a complementary perspective to established concepts such as driving forces of land-use change (Geist and Lambin 2002). While these traditional concepts mainly focus on identifying and categorising the socio-economic, institutional, and biophysical factors driving land-use changes, our analytical framework extends these by emphasising spatial and functional asymmetries within and across regions. This complements common approaches for studying land change, which primarily focus on patterns and changes in extent and intensity (Dou et al. 2021). Combining the polarisation and driving forces concepts not only allows detecting where and how polarisation occurs but also uncovering the underlying causes, such as socio-economic inequalities, policy influences, or environmental feedbacks. This combined approach could foster our understanding of land-system dynamics and support more targeted strategies for sustainable land management.

Better understanding land-system polarisation as a complex, multi-dimensional process is important for decision-making. Our results emphasise the importance of governance for polarisation as a multi-scale process, potentially crossing administrative boundaries. For example, environmental governance is increasingly confronted by telecouplings through global flows of people, goods, information, or capital (Cotta et al. 2022), such as how the European Union depends on biomass production (mainly agriculture, but also forestry) outside its own boundaries, with Latin American countries as main suppliers (Kastner et al. 2015). As interactions between telecoupled systems usually “emerge” as ungoverned processes, polarisation being one example of this, the outcomes of these interactions are often unforeseen and unintended (Eakin et al. 2014). Given the importance of polarisation for land-system sustainability, effective governance via cooperation between authorities in responding to polarisation trends is needed.

A broader challenge for addressing land-system polarisation lies in the disconnect between market policies and sustainability policies. Market liberalisation, e.g. driven by the World Trade Organisation, has led to market expansions and an increased globalisation of agrifood systems, while sustainability initiatives, e.g. promoted by the United Nations, aim at reducing the social–ecological impacts of globalisation (Primdahl and Swaffield 2010). Market mechanisms, such as price formation or capital flows, are external drivers of land change and reflect market policies that define how land-use decisions are rewarded or penalised (Debonne et al. 2021a). Consequently, land-system polarisation cannot be understood independently of the sustainability and market policy arenas, and general market structures, in which it is embedded. Hence, there is a need to reconcile market policy with social–ecological goals, a concern already articulated in the Brundtland Report (Brundtland et al. 1987).

**Fig. 5:**
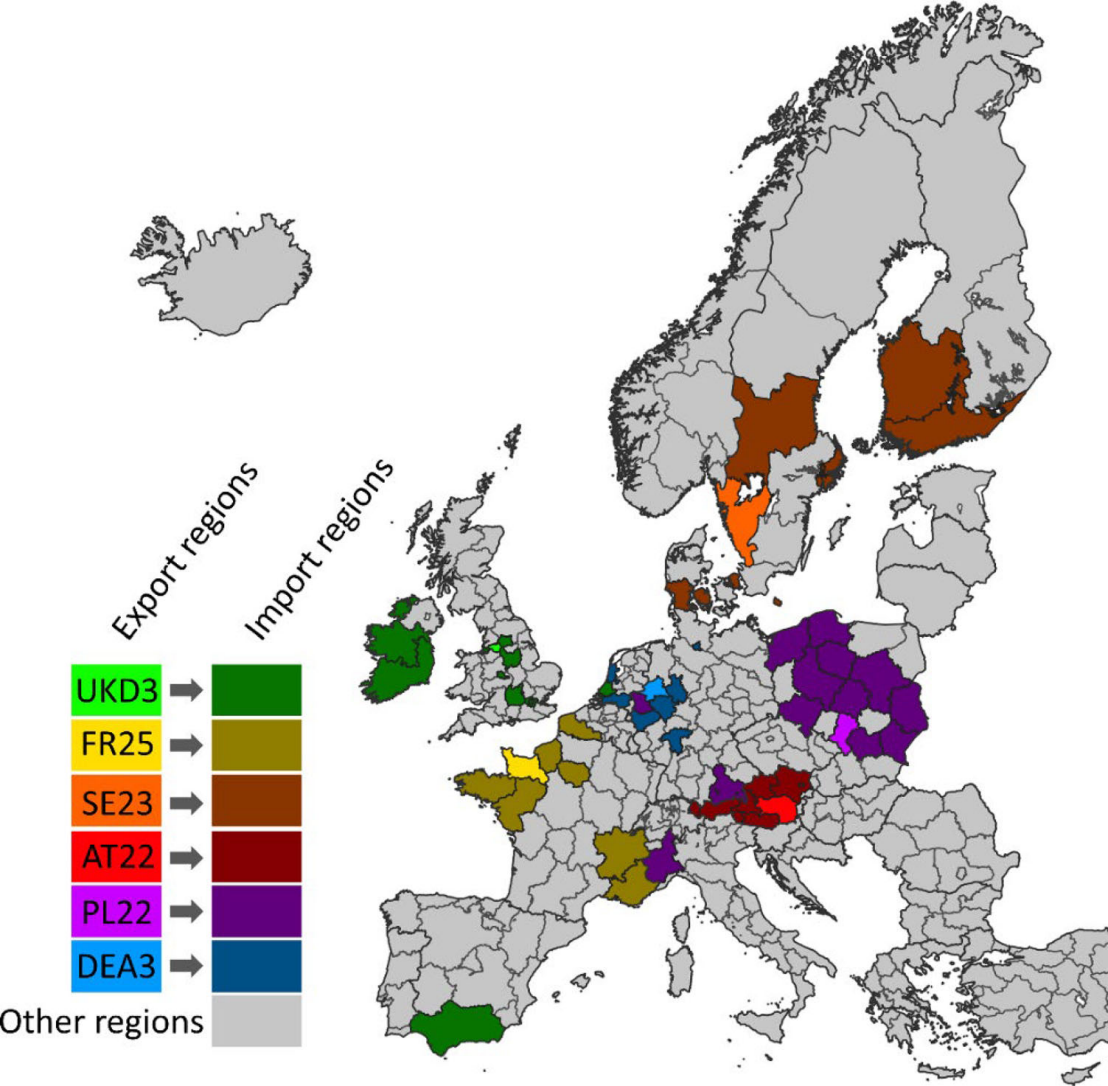
Example for spatial connections between regions in land-system polarisation. Based on EUREGIO trade data (Thissen et al. 2018; EC and JRC 2020), we selected six NUTS-2 regions as examples to visualise linkages between regions using agricultural production as an example. We define inter-regional linkages if the percentage of agricultural production value that is exported to another NUTS-2 region exceeds 2.5% of the total agricultural production value in a given NUTS2 region. We depict exporting regions in bright colours (NUTS-codes in brackets), and linked importing regions in dark colours. We hence assume that agricultural systems in regions telecoupled by trade are linked, and that polarisation can occur between these regions. For example, land changes in one region (e.g. intensification in an exporting region) can be linked to land changes in a different region (e.g. abandonment in an importing region)

We present an adaptable, scalable, and transferable framework for analysing land-system polarisation, enabling transparent, reproducible analysis of polarisation trends. Our workflow can be adjusted to the context of the analysis, in particular the choice of indicators, and allows a simple reporting which of the building blocks have been addressed and omitted. It further allows assessing drivers and outcomes of land-system polarisation for quantitative assessments of trade-offs related to the social–ecological impacts of polarisation. Moreover, linking our workflow to international trade or supply chain data (e.g. Godar et al. 2016) could help in quantifying and assigning potential spillover effects from land-system polarisation (see Meyfroidt et al. 2020), i.e. land-system polarisation in one place having impacts on land systems in other places. Many regions in Europe are linked by trade of agricultural products (Fig. 5), and changes in the production system of exporting regions (e.g. through polarisation) can have consequences for importing regions, for example, for food and fodder availability or environmental impacts (Malik et al. 2024).

Our approach has the potential to integrate different polarisation narratives under a common umbrella. Polarisation can be viewed as beneficial or harmful by different stakeholders. Providing quantitative, spatially explicit information on polarisation processes and assessments of their potential impacts on social–ecological systems could be used for guiding policy and decision-making. This information is key for assisting decision-makers in identifying potential risks and opportunities related to land-system polarisation, understanding where these are located, and developing effective strategies to mitigate them. For example, by identifying areas of high or low polarisation, decision-makers can target support for diversification or improved nutrient management strategies, or mitigating risks associated with monocultures and over-intensive farming.

Land-system polarisation also intersects with socio-economic polarisation evident in Europe, including diverging population trends (e.g. increasing concentration in and around cities versus declines mainly in rural regions), economic developments (e.g. hotspot and coldspot regions of economic prosperity), and uneven spatial and social distribution of wealth (Görmar et al. 2019). Improved knowledge of patterns, drivers, and outcomes of land-system polarisation can inform policy and management interventions, particularly in the context of the ongoing reform of the Common Agricultural Policy (CAP) as well as the Nature Restoration Regulation (NRR). Such policies and laws with their ambitious goals supporting the transition towards sustainable land use in the EU can benefit from such insights, enabling the identification of entry points for potential interventions. Ultimately, understanding polarisation is not only critical for assessing dynamics in land systems, but also for fostering resilience, environmental sustainability, and food security in the face of global challenges.

### Methodological considerations and uncertainty

While we provide a structured and innovative approach to map land-system polarisation, some limitations should be considered when interpreting the findings. First, the outcomes of applying our workflow to map land-system polarisation depend on researcher choices, assumptions, and simplifications made in operationalising our approach for a specific case study. Parameterising the five dimensions relevant for mapping land-system polarisation (“An analytical framework for mapping land-system polarisation” section) is likely to not fully capture all of its facets and characteristics, as data might be missing. However, clearly communicating the system boundaries for the analysis demonstrates what the analysis can and cannot provide (Friis and Nielsen 2017), thereby defining the realm of validity and application of the analysis. Moreover, the choice of which data to use in the analysis can have unintended consequences. For example, an indicator may be selected to represent a key characteristic of land-system polarisation due to its higher spatiotemporal resolution compared to other candidate indicators, but these other indicators may contain crucial or contradictory information that is then missed by the analysis. Sensitivity and exploratory data analyses are possible approaches to circumvent or at least document potential choice-related uncertainties in the mapping results.

Second, the application of our analytical framework strongly relies on data availability, as spatially explicit and temporally varying land-use data is needed to perform the analysis. This can impede the transferability of the workflow to regions where such data are scarce. Further, data on land-use intensity are oftentimes not available at high spatial resolution or as time series (Kuemmerle et al. 2013), for example, for pesticide application, mechanisation, or yields, which bears the limitation of omitting key dimensions of land-system polarisation. Yet, the analytical framework itself can be useful for expanding theories on land change (Meyfroidt et al. 2018), even if quantitative data are missing, as it lays out key parameters and dimensions to consider.

Third, data quality affects the outcomes of the polarisation mapping. Indicators listed in Table 1 might all be available, but their quality might differ. For example, land-use or land-cover types might be derived from classifying satellite imagery (Pflugmacher et al. 2019), nitrogen application from model-based downscaling of data at administrative unit level (Koeble et al. 2024), and yield data from agricultural statistics (Levers et al. 2016). Different data sources have different underlying methodologies and represent the given indicator with different accuracy. Such errors are propagated in analyses (Heuvelink et al. 1989), which is particularly problematic if the error remains unquantified.

Fourth, mapping polarisation trends is affected by the Modifiable Areal Unit Problem (MAUP). MAUP refers to the issue of how results of statistical analyses can be affected by the way in which geographic areas are defined and delineated, and how these areas can be modified or aggregated to produce different results. It hence represents the potential bias and limitations of spatial analysis due to the arbitrary choices of spatial units (Chen et al. 2022). The multiscale analysis allowed by our analytical framework explicitly acknowledges and addresses this scale-dependency (i.e. nested polarisation trends across spatial scales), hence limiting the potential impacts of MAUP.

Fifth, the choice of method to assess indicator trends can influence the outcomes of our polarisation mapping. We used robust slope estimates, which are less sensitive to outliers and can appropriately represent the central tendency in indicator trends. However, alternative approaches can capture different aspects of change, such as quantile-based methods of absolute changes (Kuemmerle et al. 2016). Hence, our approach potentially is likely insensitive to identify polarisation in regions where substantial absolute changes occur despite modest relative trends.

Lastly, in our analysis, we use spatially explicit indicators of crop production systems available for the EU, thereby largely neglecting the social dimension of land-system polarisation. Its representation would require including indicators such as the polarisation of attitudes and motivation of land users or managers (Swart et al. 2023), polarisation in social networks (Williams et al. 2023), or normative aspects of land-system polarisation. Unfortunately, there is a lack of spatially explicit and temporally varying data on these processes, hindering a full representation of land-system polarisation. However, in case of available and appropriate data, our analytical framework allows including indicators of the social dimension of land-system polarisation. As social polarisation processes can themselves be drivers of polarisation processes related to land use, e.g. the urban–rural divide (Bakker et al. 2021), disentangling drivers and impacts from observed patterns is challenging.

### Future research directions

Mapping land-system polarisation is a first step towards understanding polarisation processes and using this understanding to support decision-making. Several advances are needed to bridge this gap. First, identifying key factors shaping polarisation patterns is crucial for addressing its effects and implementing targeted interventions, for example, by analysing drivers and determinants of land change (Meyfroidt 2015). Second, assessing polarisation outcomes can inform policies aimed at enhancing resilience and sustainability of social–ecological systems, for example, through modelling changes in Nature’s Contributions to People in regions experiencing polarisation, enabling quantification of impacts on human well-being and the environment. Third, trade-offs arising from polarisation, for example, the supply of Nature’s Contributions to People across different polarisation profiles, could be identified through multi-criteria analysis. Fourth, evaluating spillover effects (Meyfroidt et al. 2020) from polarisation is essential for developing policies that balance economic, social, and environmental objectives. Linking our workflow to trade or supply chain data (e.g. Godar et al. 2016) could be a promising approach to quantify such spillover effects, and integrating emerging data on land-market dynamics, e.g. land tenure and ownership (Davis et al. 2025), could further improve our understanding of externally driven land-use changes. Finally, integrating the social dimension of polarisation into our analytical framework is key for a comprehensive understanding of polarisation trends. Data on social aspects of polarisation could be generated through sentiment analysis, model-based extrapolation of survey results, or agent-based modelling of stakeholder behaviour and decisions. The analytical framework and workflow presented in this paper provide a foundation upon which such future advances could be built.

## Supplementary Information

Below is the link to the electronic supplementary material.

Supplementary file1 (PDF 3563 KB)

## Data Availability

The data and code that support the findings of this study are available on request from the corresponding author [CL].
