# Supplementary material for: Conceptualising and mapping polarisation trends in land systems
Source: Ambio. 2026 Jan 24;55(7):1585–602. doi: 10.1007/s13280-025-02344-0 (PMC13230460; doi:10.1007/s13280-025-02344-0)
Supplement: Supplementary file 1 — Supplementary file1 (PDF 3563 KB) [file 13280_2025_2344_MOESM1_ESM.pdf]

Conceptualising and mapping polarisation trends in land systems

Ambio

Supporting Information

Christian Levers, Julian Helfenstein, Matthias Bürgi, Niels Debonne, Vasco Diogo, Rebekka Dossche, Felix Herzog, Yafei Li, Franziska Mohr, Rebecca Swart, Tim G. Williams, Peter H. Verburg

Corresponding Author: Dr. Christian Levers; Thünen Institute of Biodiversity, Bundesallee 65, 38116 Braunschweig, Germany; Tel: +49 531 2570 2235; Email: christian.levers@thuenen.de

Table of Contents

SUPPLEMENTARY INFORMATION: TEXT ... 2

Text S1 Detailed description of the analysis workflow ... 2

SUPPLEMENTARY INFORMATION: TABLES ... 5

Tab. S1 Overview of polarisation as a key concept in scientific fields ... 5

Tab. S2 Polarisation profiles and the number of NUTS-2 regions where they occur, using a hotspot threshold of 5% ... 6

Tab. S3 Summary of polarisation trajectories in European cropping systems ... 7

SUPPLEMENTARY INFORMATION: FIGURES ... 9

Fig. S1 Land-system polarisation as a process within social-ecological systems ... 9

Fig. S2 Individual data processing steps to map land-system plarisation, using the example of “between” and “within” indicator polarisation within observational units ... 10

Fig. S3 Spatial patterns of land-system polarisation at NUTS-2 level for crop production systems in the EU between 2000 and 2012 across different aggregation thresholds ... 11

Fig. S4 Land-system polarisation at NUTS-2 level for crop production systems in the EU between 2000 and 2012 (2.5% threshold) ... 12

Fig. S5 Land-system polarisation at NUTS-2 level for crop production systems in the EU between 2000 and 2012 (5% threshold) ... 13

Fig. S6 Land-system polarisation at NUTS-2 level for crop production systems in the EU between 2000 and 2012 (10% threshold) ... 14

SUPPLEMENTARY INFORMATION: REFERENCES ... 15

Supplementary Information: Text

Text S1 Detailed description of the analysis workflow

In the first step, we define system boundaries within which we carry out the polarisation assessment. This includes the definition of spatial and temporal scales, land-use sector(s), key characteristics/indicators, and potential linkages between observational units, as well as the definition of the target spatial resolution for the polarisation mapping (e.g., grid cell or administrative unit). In case the analysis covers multiple spatial and temporal scales, assessments can be conducted individually per scale and combined in Step 4 for a nested polarisation assessment. Then, we prepare data for the polarisation mapping (Figure S2-A). This includes the identification and acquisition of relevant indicators for the analysis and their harmonisation (e.g., coordinate reference system, target spatial resolution). We aggregate indicators from their original raster format (1x1 km^2, Figure S2-A1) to 10x10 km^2 grid cells (Figure S2-A2), the second level of the EU nomenclature of territorial units for statistics (NUTS; Figure S2-A3), and to the country level (Figure S2-A4).

In the second step, we calculate temporal trends for each indicator at the target spatial scale over the study period (Figure S2-B). The number of time steps available largely determines the calculation of the temporal trend. We here use Theil-Sen regressions to estimate robust slopes (Sen, 1968; Theil, 1992), which can usually be applied for data with time steps $N > 5$ (Carslaw and Ropkins, 2012). For data sets with less time steps, simple absolute or relative (annual) changes between the start and end year can be calculated instead. As several suitable methods for trend calculations exist, we therefore recommend sensitivity analyses to bolster the robustness of results.

In the third step, we identify hotspots of positive and negative change by classifying temporal trends. Widely applied techniques for mapping hotspots are based on the data distribution, such as the quantile method (Kuemmerle et al., 2016) or the standard deviation method (Mehrabi and Ramankutty, 2019). For example, hotspots of in- or decrease could be identified using the top and bottom decile of the distribution of temporal trends (quantile method), or using a threshold of one standard deviation above or below the study area wide average. To ensure robustness and to account for effects of using

different thresholds or intervals, both methods allow extensive sensitivity analyses. We here map hotspots of positive and negative change (Figure S2-C) based on the top and bottom quintiles (i.e., 20%) of the respective indicator distributions (Figure S2-C1 and S2-C2).

In the last step, we overlay hotspot maps for each indicator combination (e.g., hotspots of area decrease and hotspots of intensity increase) and remove grid cells with spatially co-occurring trends (e.g., hotspot of area decrease and hotspot of intensity increase in the same grid cell) from the analysis to ensure (spatial) independence of the processes (Figures S2-D1 and D2). This affects 3.5% of all grid cells for the indicator combination "extent" and "intensity", 6.3% for "extent" and "diversity", and 4.0% for "intensity" and "diversity". For each indicator combination, we calculate area shares of hotspots of decrease (Figure S2-E1) and increase (Figure S2-E2) for observational units at the target spatial resolution. In our case, this results in the aggregation of hotspot grid cells (mapped at the 10x10 km^2 scale) to the NUTS-2 level. Based on hotspot area shares, we identify and map regions with polarisation trends based on a minimum threshold. For example, a given region could be mapped as polarising for a specific indicator combination if at least a certain area share of that region is characterised by hotspots of increase and hotspots of decrease, respectively. In our case, we define polarisation for a given NUTS-2 region if opposing processes (i.e., hotspots of increase and decrease of a given indicator combination) each have an area share of at least 5% (Figure S2-F1 for within-indicator and Figure S2-F2 for between-indicator polarisation). This approach allows an extensive sensitivity analysis, e.g., by choosing more liberal (e.g., 2.5%) or conservative (e.g., 10%) thresholds. Importantly, bi-directional relationships between hotspots of change can occur, as polarisation can be characterised by hotspots of increase (Indicator A) and decrease (Indicator B), but also by hotspots on decrease (Indicator A) and increase (Indicator B). Overlaying polarisation maps for each indicator combination (Figure S2-F3) results in the final polarisation map. This allows identifying regions characterised by various co-occurring polarisation trends (e.g., within-indicator AND between-indicator polarisation), accounting for its complexity given potentially multiple co-occurring trends within a region.

To identify nested polarisation patterns, polarisation results (cf. Step 4) of individual temporal and/or spatial scales can be overlaid. For example, a landscape (e.g., a 10x10 km^2 grid cell) can be identified as polarising for a given indicator combination (e.g., area decrease and intensity increase), which can be embedded in an administrative region (e.g., NUTS-2 region) and/or a country that is polarising for the same indicator combination. In this case, landscape-scale polarisation would be representative for a broad-scale trend. If the administrative region and country do not indicate polarisation trends (or different ones), the observed landscape-scale polarisation would be indicative for a local trend.

Supplementary Information: Tables

Tab. S1 Overview of polarisation as a key concept in scientific fields

Scientific field	Domain	Description
Physics	Electromagnetism	Direction of oscillation of an electromagnetic wave.
	Optics	Alignment of the electric field vector of light waves.
Biophysics	Biomolecular science	Separation of electrical charges across the molecular structure, affecting its interactions with other molecules and its biological function.
Chemistry	Molecular chemistry	Distortion of electron clouds within a molecule, causing it to have an uneven distribution of charge, which responsible for many of the properties of molecules, including their polarity, reactivity, and solubility.
Biology	Cell biology	Asymmetric distribution of cellular components during cell division and differentiation, which is critical for the formation of complex tissues and organs, and is essential for proper development and function of organisms.
	Molecular genetics	Separation of charged molecules, such as DNA and RNA, in an electric field, affecting their migration and separation.
Geology	Geophysics	Detect and map the subsurface structures and properties of rocks and minerals. This is achieved through the use of techniques such as electrical resistivity, electromagnetic induction, and seismic reflection, which rely on the polarisation properties of the Earth's materials.
Materials science	Materials science	Characterize the electrical, optical, and magnetic properties of materials. This includes the study of ferroelectric and ferromagnetic materials, which exhibit polarisation behaviour that is crucial for their applications in technologies such as memory storage and sensors.
Psychology	Social psychology	Tendency of groups to become more extreme in their attitudes and beliefs over time. This can lead to opposition between groups with opposing views, and can have significant social and political consequences.
Political science	Politics	Growing ideological divide between political parties and groups. This can lead to increased partisanship, gridlock, and opposition in the political system, with implications for democratic governance and policy-making.
Engineering	Electrical engineering	Describe the alignment and orientation of electric fields in electronic devices, e.g. capacitors and antennas, which affect quality and efficiency of signal transmission.
Astronomy	Cosmology	Measure the polarisation properties of the cosmic microwave background radiation, and to study the magnetic fields of galaxies and interstellar gas.
Mathematics	Algebraic geometry	Process of decomposing multivariable polynomials into simpler terms.
	Tensor calculus	Process of transforming vectors and tensors to better understand their properties.
Environmental science	Atmospheric science	Absorption and reflection of solar radiation by the Earth's atmosphere and surface, which is responsible for the greenhouse effect, the primary driver of global warming.
	Environmental pollution	Separation of pollutants from water or air using polar membranes or adsorbents. It can also be used to identify and quantify sources of pollution.

Computer science	Machine learning	Process of separating data into distinct categories, affecting accuracy and reliability of machine learning algorithms.
	Computer vision	Process of image separation (foreground and background).
Linguistics	Semantics	Organization of meaning into opposing pairs of concepts, such as hot/cold, good/bad, and positive/negative.
Economics	Income inequality	Growing divide between high- and low-income households. This can have significant implications for social mobility, economic growth, and political stability.
Sociology	Social inequality	Growing divide between different social groups, such as race, gender, and class. This can lead to social stratification and marginalization, and can have significant implications for social justice and human rights.
Neuroscience	Brain function	Separation of electrical charges across the cell membrane of neurons. This process is critical for the transmission of electrical signals in the brain, and is responsible for many of the properties of neural networks.
Pharmacology	Drug action	Separation of electrical charges across the cell membrane of target cells, which affects binding and efficacy of drugs.
Musicology	Musical harmony	Organization of sound into opposing pairs of pitches, such as consonance/dissonance and tension/release, affecting the emotional and aesthetic impact of music on the listener.
Anthropology	Cultural anthropology	Separation of cultural practices and beliefs into opposing pairs, such as tradition/innovation and individualism/ collectivism. This can have significant implications for cultural change and identity formation.
History	Political history	Growing divide between different political factions and ideologies. This can lead to social conflict, violence, and revolution, significantly affecting the course of history.
Philosophy	Ethics	Separation of moral values into opposing pairs, such as right/wrong and good/evil, affecting the development of ethical systems and the interpretation of moral dilemmas.
Education		Increasing divide between different educational institutions and curricula, leading to social inequality and division.

Tab. S2 Polarisation profiles and the number of NUTS-2 regions where they occur, using a hotspot threshold of 5%

Polarisation profile	# NUTS-2 regions
No polarisation	33
Between	16
Within-Spat	8
Spat	9
Between-within	87
Between-Spat	7
Within-Spat	11
All	90
Total	261
Polarisation [#]	228
Polarisation [%]	87.36

Tab. S3 Summary of polarisation trajectories in European cropping systems. Each row provides examples and a hypothesized mechanism for distinct combinations of changes in extent, intensity, and composition for two spatially disjoint units

Indicator trajectories	Spatial unit 1	Spatial unit 2	Potential mechanism	Examples
Extent (↑) & Intensity (↓)	Increasing extent: New, often marginal land is converted to cropland for production (e.g., olive groves).	Decreasing intensity: Traditional olive groves (e.g.in heritage landscapes) shift towards lower fertiliser and herbicide inputs, responding to agri-environmental measures	Land availability vs. regulatory pressure: Expansion onto new, marginal land to increase production occurs alongside de-intensification of existing land to reach environmental goals, for example as a result of policy that effectively zones land for different functions.	(Guzmán et al., 2022; Morgado et al., 2022)
Extent (↓) & Intensity (↑)	Decreasing Extent: High-productive cropland is lost to urbanisation or infrastructure.	Increasing Intensity: Remaining farms intensify production through higher N inputs to maximise yields.	Land competition and economic optimisation: Increased land rent near urban regions leads to the decline of farming at the urban fringe, while intensification of farming occurs in more remote areas or on remaining urban-adjacent land to meet rising demand or support higher costs.	(Beckers et al., 2020; Delbecq and Florax, 2010)
Composition (↑) & Intensity (↓)	Increasing simplification: Large-scale farms simplify crop rotations to streamline logistics and achieve economies of scale.	Decreasing intensity: Farms significantly cut fertiliser use to reduce costs or comply with regulations, resulting in less input-intensive systems.	Divergent economic and regulatory pressures: Different farm types face different constraints with large farms specialising for efficiency and marginal farms cutting inputs, leading to lower-intensity systems.	(Verburg et al., 2022)
Composition (↓) & Intensity (↑)	Decreasing simplification: Farms adopt complex rotations with legumes and cover crops to improve soil health and meet demand for sustainable food.	Increasing intensity: Farms focus on high-value, input-responsive commodities and increase fertilizer use to push yields to their maximum.	Divergent market integration and farm strategies: This is a bifurcation of the sector with some farms oriented towards quality and resilience, others towards increased outputs and profits.	(Verkuil et al., 2024)
Composition (↑) & Extent (↓)	Increasing simplification: Agricapital invests in large land parcels to create specialized operations focused on a single high-profit commodity.	Decreasing extent: On remote or poor-quality land, small-scale farming becomes non-viable and land is abandoned.	Economic marginalisation and economies of scale: Economies of scale favour large, specialised farms on the highly productive land, while small farms on marginal land are abandoned.	(Dogaru et al., 2024)

Composition (↓) & Extent (↑)	Decreasing simplification: Farms close to urban centres diversify into high-value vegetables and niche products for direct sales.	Increasing extent: To meet global demand for commodities, cropland expands into new areas to increase production.	Proximity to contrasting markets: The type of market access affects production systems with proximity to urban consumers driving diversified systems, while other systems expand to produce a few commodity crops.	(van Vliet, 2019)
Extent (↑) & Extent (↓)	Increasing extent: Arable land expands in areas with favourable production conditions or where land is available.	Decreasing extent: Arable land is abandoned in marginal areas with unfavourable conditions for crop production.	Profit maximisation vs. economic marginalisation: Arable farming on larger productive areas increases production and profits, while less competitive farms in marginal regions lose viability, leading to abandonment.	(Hatna and Bakker, 2011)
Intensity (↑) & Intensity (↓)	Increasing Intensity: Large arable farms in productive regions adopt high-input, mechanised production systems to maximise yields.	Decreasing intensity: Small or marginal farms reduce fertiliser and pesticide use, shifting toward extensive or low-input systems.	Capital, technology, and market access: Large farms in fertile regions intensify production using machinery, fertilisers, and high-yield varieties, supported by market integration and profitability, while smaller or remote farms remain low-input due to limited resources and weak economic incentives.	(Plieninger et al., 2016)
Composition (↑) & Composition (↓)	Increasing simplification: Larger farms specialise their production to achieve economies of scale and supply larger markets and commodity channels.	Decreasing simplification: Smaller farms diversify and are entirely or partly connected to local markets and marketing channels.	Market integration and consumer proximity: Access to global commodity markets drives specialisation in some farms, while access to local urban markets, direct sales, and niche products supports diversification strategies.	(de Roest et al., 2018)

Supplementary Information: Figures

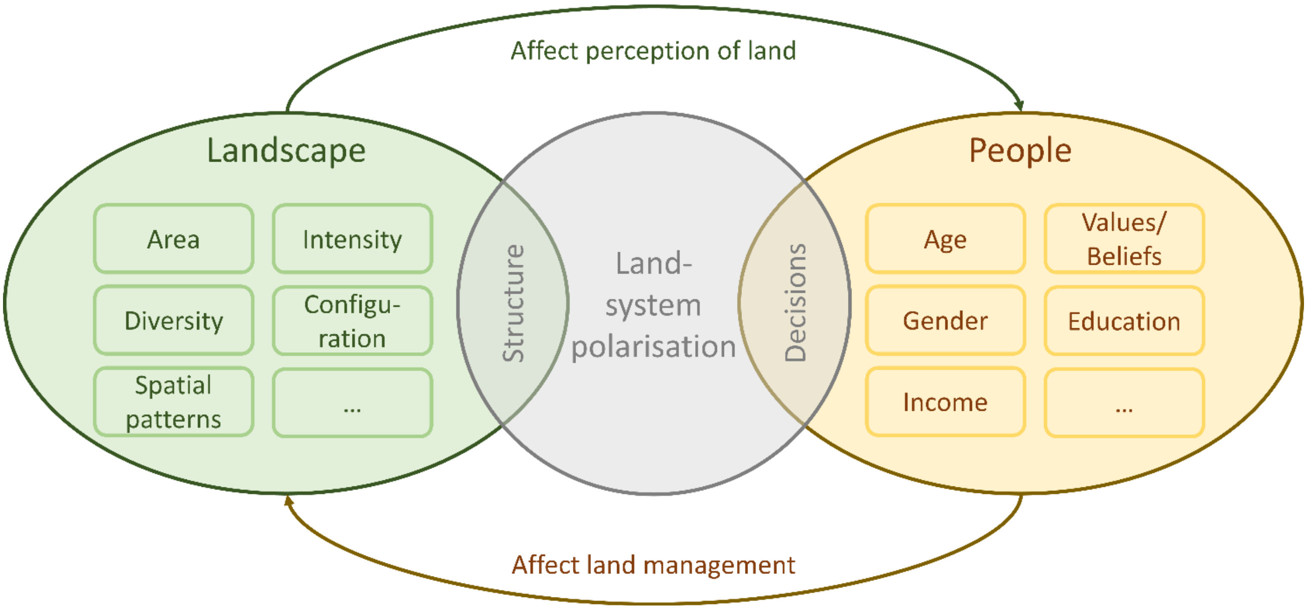

Fig. S1 Land-system polarisation as a process within social-ecological systems. Polarisation can occur in the ecological domain as tangible and mappable landscape changes, but also in the social domain through socio-economic, political, or cultural changes. Importantly, polarisation in the social domain can affect land management decisions and hence polarisation outcomes observable in the landscape. In turn, polarisation trends observable in the landscape can affect people's perception of land and can trigger polarisation in the social domain

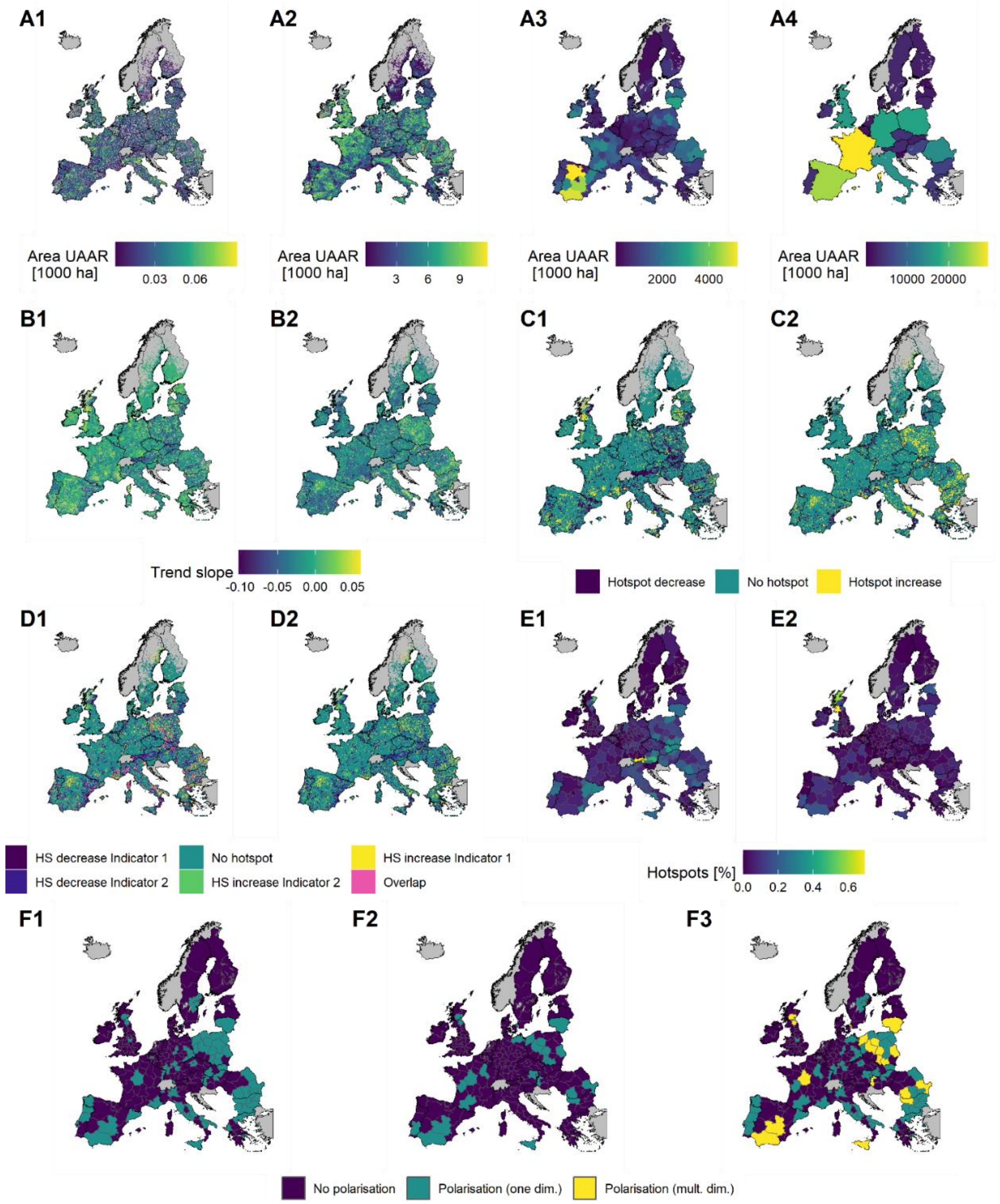

Fig. S2 Individual data processing steps to map land-system plarisation, using the example of "between" and "within" indicator polarisation within observational units. Panel A: indicator aggregation for "utilised agricultural area" from its original resolution (1x1 km^2, A1) to 10x10 km^2 (A2), NUTS-2 (A3) and country level (A4). Panel B: trend slopes between 2000 and 2012 for "utilised agricultural area" (B1) and "Shannon crop diversity" (B2) based on Theil-Sen trend regressions. Panel C: hotspots of increase and decrease for "utilised agricultural area" (C1) and "Shannon crop diversity" (C2), based on the top 20% of the respective data distributions. Panel D: overlay of hotspot maps (Panels C1 and C2) for "utilised agricultural area" and "Shannon crop diversity", with (D1) and without (D2) grid cells that overlap between both indicators. Panel E: area share of hotspots of decrease (E1) and increase (E2) per NUTS-2 region for "utilised agricultural area". Panel F: polarisation per NUTS-2 region based on "utilised agricultural area" and "Shannon crop diversity" ("between indicator" polarisation; F1) and "utilised agricultural area" ("within indicator" polarisation; F2). The overlay of both maps (F3) highlights regions where no, one, or multiple polarisation trends have been detected

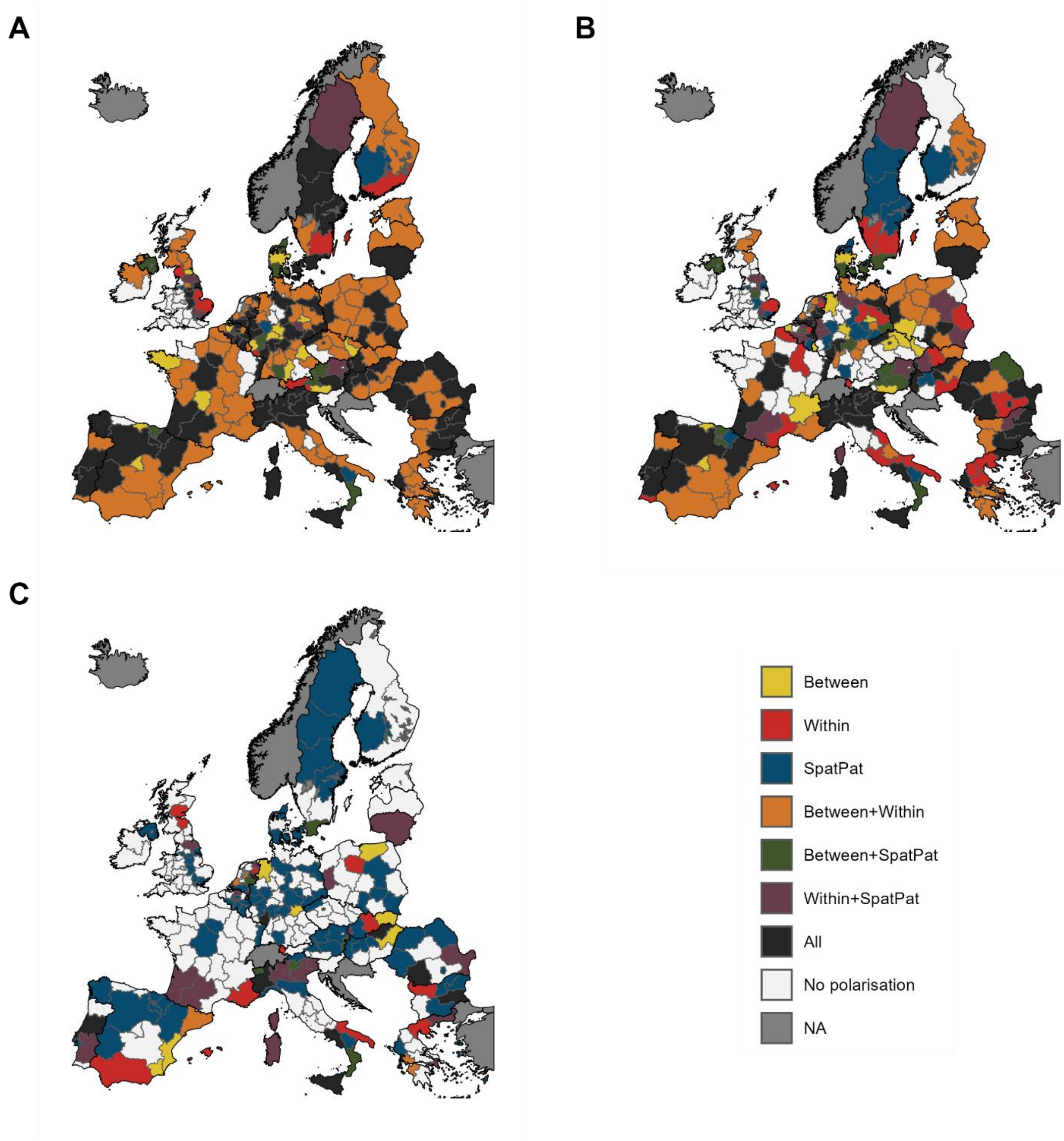

Fig. S3 Spatial patterns of land-system polarisation at NUTS-2 level for crop production systems in the EU between 2000 and 2012 across different aggregation thresholds. Panel A shows polarisation patterns based on a 2.5% threshold regarding hotspots of change (i.e., hotspots of in- and decrease each have to cover at least 2.5% of the area of a NUTS-2 region to indicate polarisation), Panel B shows polarisation patterns based on a 5% threshold, Panel C polarisation patterns based on a 10% threshold

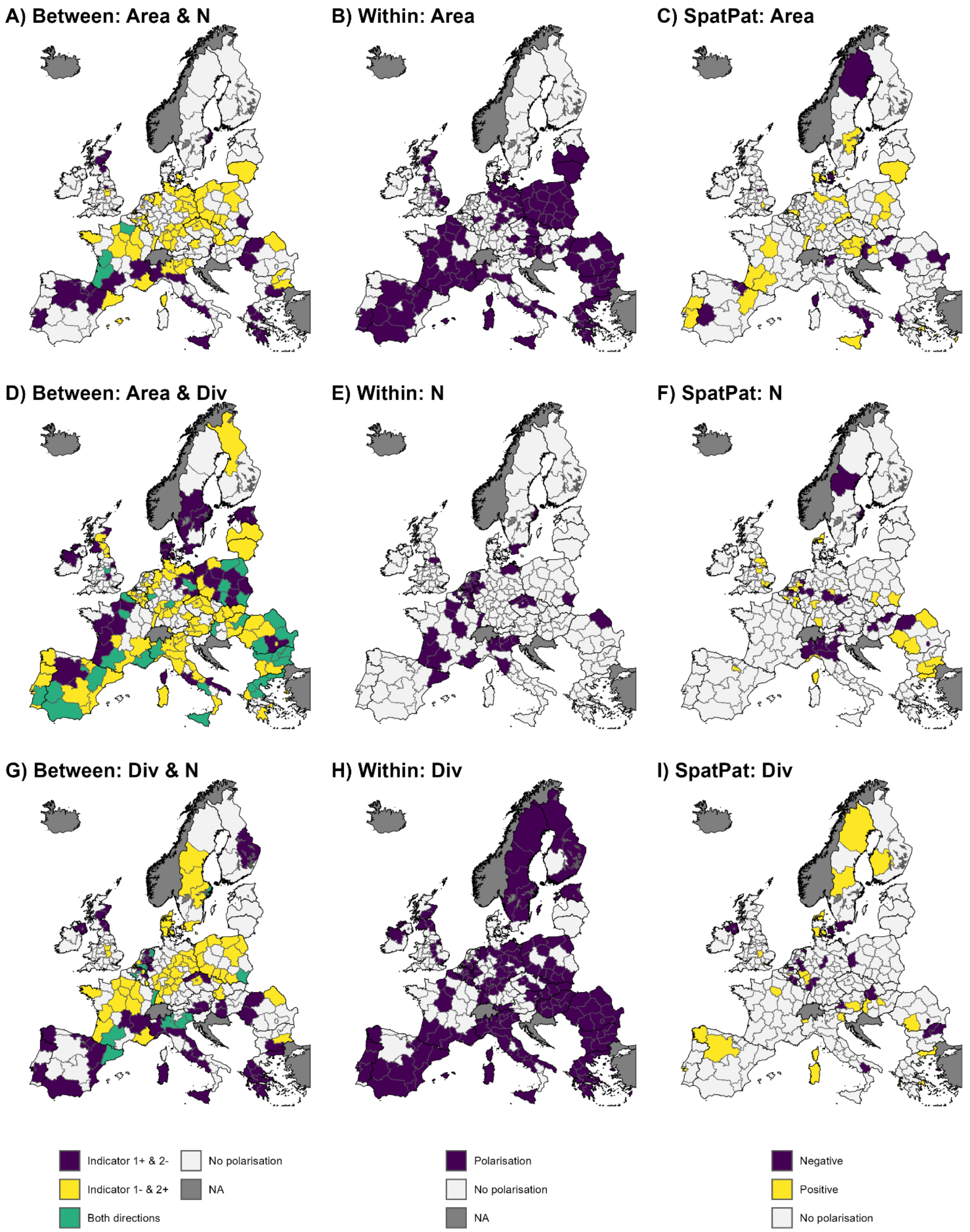

Fig. S4 Land-system polarisation at NUTS-2 level for crop production systems in the EU between 2000 and 2012 (2.5% threshold). Columns show "between" (left column), "within" (centre column), and "spatial pattern" (right column) polarisation, for the three indicators "cropland area", "nitrogen input", and "Shannon crop diversity"

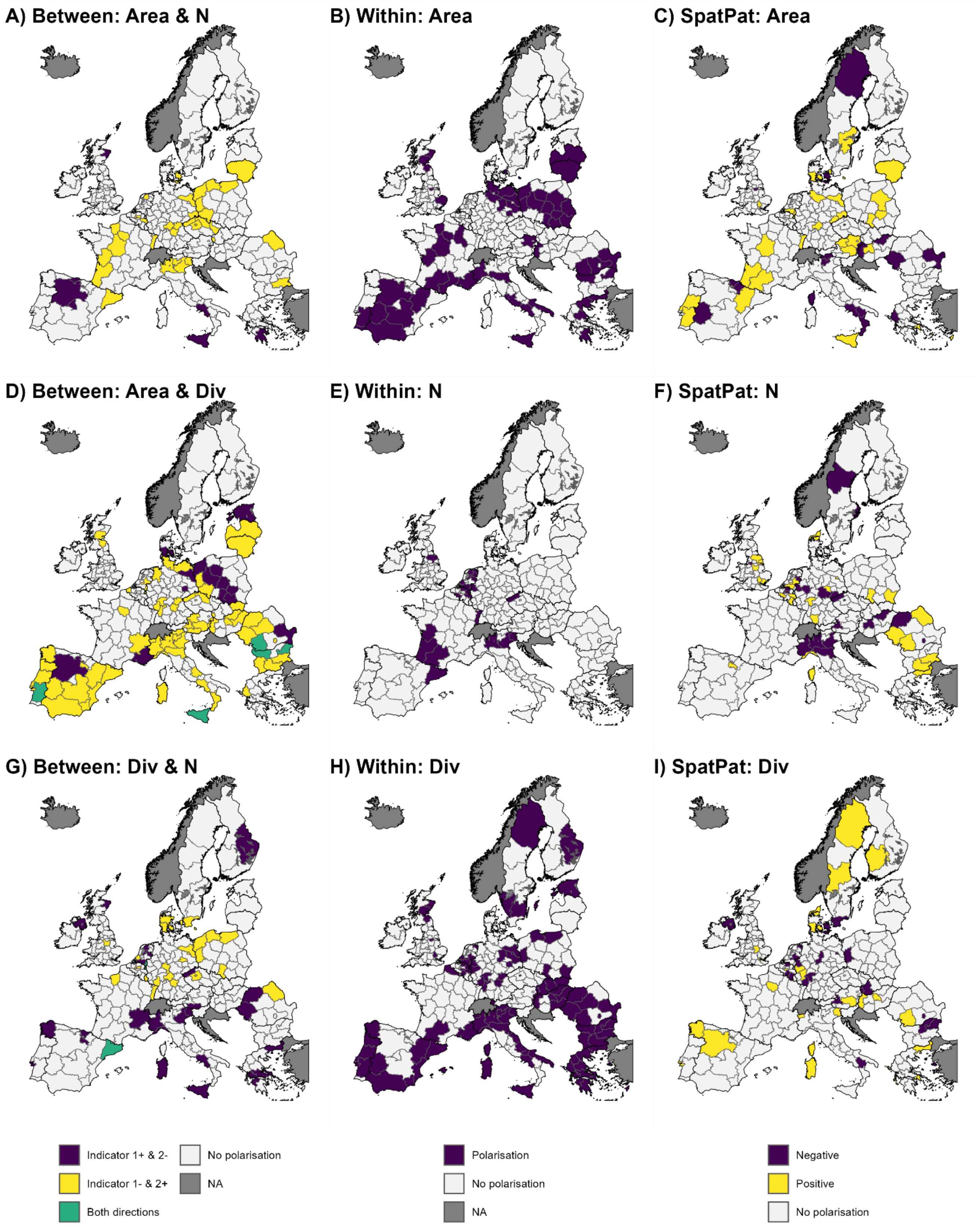

Fig. S5 Land-system polarisation at NUTS-2 level for crop production systems in the EU between 2000 and 2012 (5% threshold). Columns show "between" (left column), "within" (centre column), and "spatial pattern" (right column) polarisation, for the three indicators "cropland area", "nitrogen input", and "Shannon crop diversity"

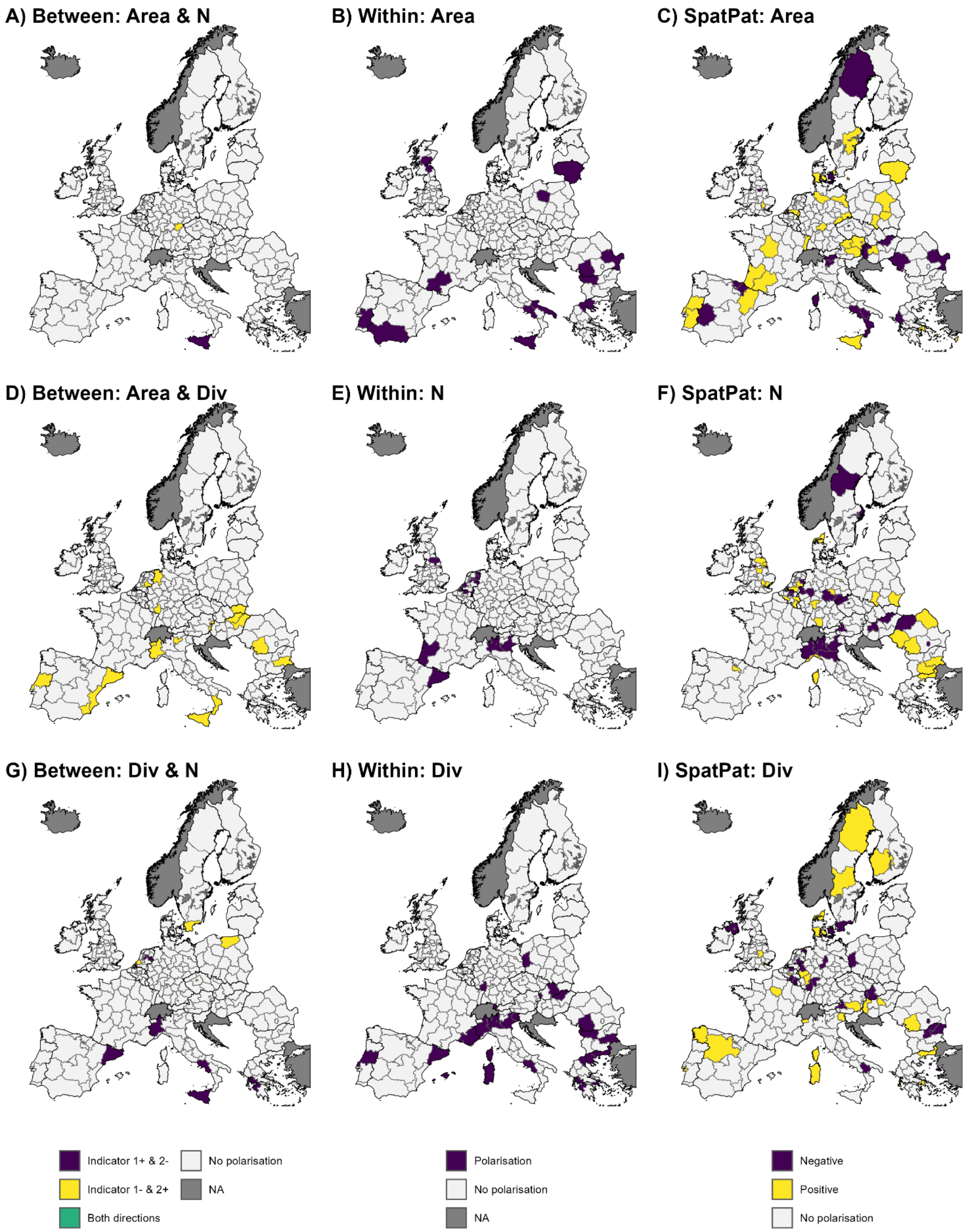

Fig. S6 Land-system polarisation at NUTS-2 level for crop production systems in the EU between 2000 and 2012 (10% threshold). Columns show “between” (left column), “within” (centre column), and “spatial pattern” (right column) polarisation, for the three indicators “cropland area”, “nitrogen input”, and “Shannon crop diversity”

Supplementary Information: References

Beckers, V., Poelmans, L., Van Rompaey, A., Dendoncker, N., 2020. The impact of urbanization on agricultural dynamics: a case study in Belgium. J. Land Use Sci. 15, 626–643. https://doi.org/10.1080/1747423X.2020.1769211

Carslaw, D.C., Ropkins, K., 2012. openair — An R package for air quality data analysis. Environ. Model. Softw. 27–28, 52–61. https://doi.org/10.1016/j.envsoft.2011.09.008

de Roest, K., Ferrari, P., Knickel, K., 2018. Specialisation and economies of scale or diversification and economies of scope? Assessing different agricultural development pathways. J. Rural Stud. 59, 222–231. https://doi.org/10.1016/j.jrurstud.2017.04.013

Delbecq, B.A., Florax, R.J.G.M., 2010. Farmland Allocation along the Rural-Urban Gradient: The Impacts of Urbanization and Urban Sprawl. Selected Paper. https://doi.org/10.22004/ag.econ.61723

Dogaru, D., Petrisor, A.-I., Angearu, C.-V., Lupu, L., Bălteanu, D., 2024. Land Governance and Fragmentation Patterns of Agricultural Land Use in Southern Romania during 1990–2020. Land 13, 1084. https://doi.org/10.3390/land13071084

Guzmán, G., Boumahdi, A., Gómez, J.A., 2022. Expansion of olive orchards and their impact on the cultivation and landscape through a case study in the countryside of Cordoba (Spain). Land Use Policy 116, 106065. https://doi.org/10.1016/j.landusepol.2022.106065

Hatna, E., Bakker, M.M., 2011. Abandonment and Expansion of Arable Land in Europe. Ecosystems 14, 720–731. https://doi.org/10.1007/s10021-011-9441-y

Kuemmerle, T., Levers, C., Erb, K., Estel, S., Jepsen, M.R., Müller, D., Plutzar, C., Stürck, J., Verkerk, P.J., Verburg, P.H., Reenberg, A., 2016. Hotspots of land-use change in Europe. Environ. Res. Lett. 11, 064020. https://doi.org/10.1088/1748-9326/11/6/064020

Mehrabi, Z., Ramankutty, N., 2019. Synchronized failure of global crop production. Nat. Ecol. Evol. 3, 780–786. https://doi.org/10.1038/s41559-019-0862-x

Morgado, R., Ribeiro, P.F., Santos, J.L., Rego, F., Beja, P., Moreira, F., 2022. Drivers of irrigated olive grove expansion in Mediterranean landscapes and associated biodiversity impacts. Landsc. Urban Plan. 225, 104429. https://doi.org/10.1016/j.landurbplan.2022.104429

Plieninger, T., Draux, H., Fagerholm, N., Bieling, C., Bürgi, M., Kizos, T., Kuemmerle, T., Primdahl, J., Verburg, P.H., 2016. The driving forces of landscape change in Europe: A systematic review of the evidence. Land Use Policy 57, 204–214. https://doi.org/10.1016/j.landusepol.2016.04.040

Sen, P.K., 1968. Estimates of the Regression Coefficient Based on Kendall's Tau. J. Am. Stat. Assoc. 63, 1379–1389. https://doi.org/10.1080/01621459.1968.10480934

Theil, H., 1992. A Rank-Invariant Method of Linear and Polynomial Regression Analysis, in: Raj, B., Koerts, J. (Eds.), Henri Theil's Contributions to Economics and Econometrics: Econometric Theory and Methodology, Advanced Studies in Theoretical and Applied Econometrics. Springer Netherlands, Dordrecht, pp. 345–381. https://doi.org/10.1007/978-94-011-2546-8_20

van Vliet, J., 2019. Direct and indirect loss of natural area from urban expansion. Nat. Sustain. 2, 755–763. https://doi.org/10.1038/s41893-019-0340-0

Verburg, R.W., Verberne, E., Negro, S.O., 2022. Accelerating the transition towards sustainable agriculture: The case of organic dairy farming in the Netherlands. Agric. Syst. 198, 103368. https://doi.org/10.1016/j.agsy.2022.103368

Verkuil, L.A., Verburg, P.H., Levers, C., Stratton, A.E., Schulp, C.J.E., 2024. Bright spots of agroecology in the Netherlands: A spatial analysis of agroecological practices and income stability. Agric. Syst. 220, 104086. https://doi.org/10.1016/j.agsy.2024.104086
